# Molecular mechanism of ligand recognition by membrane transport protein, Mhp1

**DOI:** 10.15252/embj.201387557

**Published:** 2014-06-21

**Authors:** Katie J Simmons, Scott M Jackson, Florian Brueckner, Simon G Patching, Oliver Beckstein, Ekaterina Ivanova, Tian Geng, Simone Weyand, David Drew, Joseph Lanigan, David J Sharples, Mark SP Sansom, So Iwata, Colin WG Fishwick, A Peter Johnson, Alexander D Cameron, Peter JF Henderson

**Affiliations:** 1 School of Chemistry and Astbury Centre for Structural Molecular Biology, University of LeedsLeeds, UK; 2 School of Biomedical Sciences and Astbury Centre for Structural Molecular Biology, University of LeedsLeeds, UK; 3 Membrane Protein Laboratory, Diamond Light Source, Harwell Science and Innovation CampusChilton, Didcot, UK; 4 Division of Molecular Biosciences, Membrane Protein Crystallography Group, Imperial CollegeLondon, UK; 5 Rutherford Appleton Laboratory, Research Complex at HarwellHarwell, Oxford, Didcot, UK; 6 Department of Physics, Arizona State UniversityTempe, AZ, USA; 7 Department of Biochemistry, University of OxfordOxford, UK; 8 School of Life Sciences, University of WarwickCoventry, UK

**Keywords:** five helix inverted repeat superfamily, hydantoin, membrane transport, Mhp1, molecular recognition, nucleobase-cation-symport, NCS1, family

## Abstract

The hydantoin transporter Mhp1 is a sodium-coupled secondary active transport protein of the nucleobase-cation-symport family and a member of the widespread 5-helix inverted repeat superfamily of transporters. The structure of Mhp1 was previously solved in three different conformations providing insight into the molecular basis of the alternating access mechanism. Here, we elucidate detailed events of substrate binding, through a combination of crystallography, molecular dynamics, site-directed mutagenesis, biochemical/biophysical assays, and the design and synthesis of novel ligands. We show precisely where 5-substituted hydantoin substrates bind in an extended configuration at the interface of the bundle and hash domains. They are recognised through hydrogen bonds to the hydantoin moiety and the complementarity of the 5-substituent for a hydrophobic pocket in the protein. Furthermore, we describe a novel structure of an intermediate state of the protein with the external thin gate locked open by an inhibitor, 5-(2-naphthylmethyl)-L-hydantoin, which becomes a substrate when leucine 363 is changed to an alanine. We deduce the molecular events that underlie acquisition and transport of a ligand by Mhp1.

## Introduction

Mhp1 from the Gram-positive *Microbacterium liquefaciens* is an integral membrane protein that mediates the Na^+^-dependent binding and uptake of 5-aryl-substituted hydantoins (Suzuki & Henderson, 2006; Weyand *et al*, 2008). Hydantoins are important compounds in salvage pathways for nitrogen balance in yeasts and plants and are particularly interesting commercially for the synthesis of chiral amino acids (Bommarius *et al*, 1998; Altenbuchner *et al*, 2001; Suzuki *et al*, 2005). Mhp1 belongs to the nucleobase-cation-symport-1, NCS1, family of secondary active transporters (Weyand *et al*, 2008) found widely in bacteria (de Koning & Diallinas, 2000), archaea (Ma *et al*, 2013), fungi (Pantazopoulou & Diallinas, 2007) and plants (Mourad *et al*, 2012; Witz *et al*, 2012; Schein *et al*, 2013). Transporters from the NCS1 family are also important in the toxicity of the antifungal agent, 5-flucytosine (Paluszynski *et al*, 2006), and mutations in the proteins can lead to drug resistance (Chen *et al*, 2011). Mhp1 is an excellent model system for elucidating how substrates or inhibitors, including drugs, are recognised at the molecular level and then taken up into cells by members of the NCS1 transporter family.

Mhp1 is of more general significance because it is also structurally homologous to other proteins in different subfamilies of a superfamily of secondary transporters (Wong *et al*, 2012). These include LeuT (Yamashita *et al*, 2005) of the neurotransmitter-sodium-symport family (NSS), vSGLT of the solute-sodium-symporter family (SSS) (Faham *et al*, 2008), BetP (Ressl *et al*, 2009) and CaiT (Schulze *et al*, 2010; Tang *et al*, 2010) of the betaine-carnitine-choline family (BCCT), and AdiC (Fang *et al*, 2009; Gao *et al*, 2009; Kowalczyk *et al*, 2011), ApcT (Shaffer *et al*, 2009) and GadC (Ma *et al*, 2012) of the amino acid-polyamine-organocation family (APC). Members of the NSS, SSS and APC families play important roles in human physiology, being responsible for the accumulation of molecules such as neurotransmitters, sugars, amino acids and drugs into cells (Gether *et al*, 2006; Broer & Palacin, 2011; Wright, 2013). As for Mhp1, transport in LeuT, BetP and vSGLT is driven by the cotranslocation of sodium ions (Abramson & Wright, 2009; Perez & Ziegler, 2013), but the superfamily also contains many examples of proton-coupled symporters or antiporters. The superfamily has been termed the 5-helix inverted repeat transporter superfamily (5HIRT), as each protein has a core of ten transmembrane helices with pseudo twofold symmetry relating repeats of five helices (Abramson & Wright, 2009). These proteins, like other secondary transporters, utilise a mechanism described by the “alternating access” model of membrane transport (Jardetzky, 1966) with their similar structures implying commonalities of mechanism (Abramson & Wright, 2009; Krishnamurthy *et al*, 2009; Forrest *et al*, 2011; Shi, 2013). In this model, conformational changes to the protein alternately expose the substrate-binding site to the outside or the inside of the cell. In switching between these two states, the protein adopts one or more intermediate states, at least one of which must be occluded. Mhp1 was the first secondary transporter for which an outward, an inward and an occluded state was characterised crystallographically, and this has provided much useful insight into the mechanism of alternating access (Weyand *et al*, 2008; Shimamura *et al*, 2010; Weyand *et al*, 2011; Shi, 2013). Mhp1 was also used to model the outward-facing form of the human Na^+^-glucose cotransporter in combination with the inward-facing form of vSGLT (Sala-Rabanal *et al*, 2012).

The structure of Mhp1 comprises twelve transmembrane helices (TMHs), which include the ten core TMHs characteristic of the superfamily with an additional two at the C-terminus (Weyand *et al*, 2008). The core can be divided into two motifs, a bundle motif (TMHs 1, 2, 6 and 7) and a hash motif (TMHs 3, 4, 8 and 9) (Shimamura *et al*, 2010). Two additional helices, TMHs 5 and 10, link the bundle with the hash motif and the hash motif to the C-terminal TMHs, respectively. Based upon structural data, the currently accepted mechanism (Shimamura *et al*, 2010) involves substrate binding to the outward-facing conformation of the transporter in a cavity between the bundle and hash domains. The N-terminal part of TMH10 then folds over the substrate to occlude it in the binding site (Weyand *et al*, 2008). Subsequently, the protein can switch to the inward-facing conformation by a predominantly rigid body rotation of the hash domain relative to the bundle domain (Shimamura *et al*, 2010). For sodium-coupled transporters, the conformational changes have been described in terms of the opening and closing of thick and thin gates (Krishnamurthy *et al*, 2009); in Mhp1, TMHs 5 and 10 correspond to the intra- and extracellular thin gates, respectively, and the rotation of the hash motif relative to the bundle corresponds to the movement of the thick gate. The binding site for sodium ions is located at the interface between the bundle and hash motifs and is only fully formed in the outward-facing structures.

Although the previously solved structure of Mhp1 in complex with L-5-benzylhydantoin (L-BH) was sufficient to reveal where the substrate binds and how the movement of TMH10 is able to occlude the binding site, the details of these events were obscure. Here, we elucidate the structural basis of ligand binding to Mhp1 and the consequent movements of individual amino acids and overall conformational changes using a combination of X-ray crystallography, molecular dynamics, site-directed mutagenesis, ligand design, synthetic chemistry and biochemical/biophysical assays. We characterise L-5-(2-naphthylmethyl)hydantoin (NMH) as a non-transported inhibitor of Mhp1 and show how the structure of the Mhp1-NMH complex provides insight into the role of TMH10 in transport.

## Results

### Crystal structures of Mhp1-hydantoin complexes

**Figure 1:**
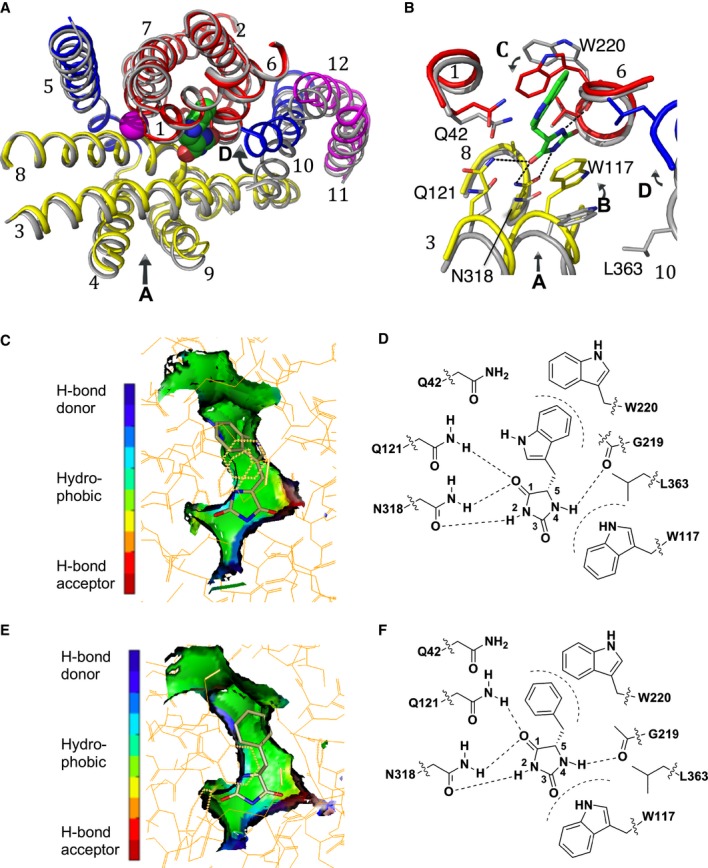
Binding of substrates in Mhp1 A, B Superposition of the outward-open structure (PDB code 2JLN) onto the IMH-bound structure, optimised using the bundle helices. The IMH structure is shown with the bundle in red, the hash motif in yellow, TMHs 5 and 10 in blue and the C-terminal helices in magenta. The outward-open structure is shown in grey. The L-IMH (green spheres) and sodium ion (magenta) bind between the hash and bundle motifs. (A) shows an overview of all helices and (B) a close up. The arrows show the main conformational changes that occur upon L-IMH binding. Arrow A: the hash motif rotates towards the bundle with the C-terminal helices partially following. Arrows B and C: Trp117 and Trp220 rotate towards the hydantoin moiety and the 5-indole substituent, respectively, of L-IMH. Arrow D: TMH10 flexes and packs over the IMH. C The extended form of L-IMH in the binding site illustrated using the SPROUT format (Materials and Methods and Supplementary Methods) to show the indole moiety in a hydrophobic pocket (green). D Schematic of interactions made between L-IMH and the protein. Possible hydrogen bonds are indicated by straight dashed lines and hydrophobic interactions by curved dashed lines. E The extended form of L-BH is oriented similarly to L-IMH with its benzyl moiety in the hydrophobic pocket. F Schematic of the interactions made by L-BH with Mhp1. Data information: In (C and E) green represents regions where a hydrophobic interaction can be made, blue represents regions containing hydrogen-bond donor atoms, and red represents regions containing hydrogen-bond acceptor atoms.

The structure of Mhp1 in complex with L-5-(1H-indol-3-ylmethyl)hydantoin (L-IMH) was determined at 3.4 Å resolution (Table1). The L-IMH ligand is clearly defined in the electron density, residing between the bundle and hash motifs (Fig1A and B, and Supplementary Fig S1A). Although the position of L-IMH is similar to that of L-BH in the previously reported structure, the higher-resolution and better quality maps associated with the new structure enabled significant improvement in our understanding of ligand binding. L-IMH binds in a more extended conformation than was previously modelled for L-BH with the hydantoin ring reoriented by 180°. The hydantoin moiety mainly interacts with residues on the hash motif. It lies approximately parallel to the indole ring of Trp117 such that the two aromatic groups form a face-to-face π-stacking interaction and appears to be oriented by hydrogen bonding interactions with Asn318, Gln121 and Gly219 (Fig1B–D). This latter interaction with Gly219 at the breakpoint of TMH6 on the bundle was not apparent previously.

**Table 1:** Statistics for the X-ray diffraction analyses

	L-IMH	L-BH	BVH	D/L-NMH
Beamline	Diamond I02	Diamond I03	Diamond I03	Diamond I04
Wavelength (Å)	0.9795	0.97630	0.91910	0.9795
Resolution (Å)^a^	3.4 (3.49-3.4)	3.8 (3.87-3.8)	3.7 (3.76-3.7)	3.7 (3.8-3.7)
Space group	P2_1_2_1_2_1_	P2_1_2_1_2_1_	P2_1_2_1_2_1_	P2_1_2_1_2_1_
Cell dimensions (Å)	87.4 106.4 100.6	95.6 106.7 107.9	90.0 107.3 109.3	87.6 106.9 106.8
No. measured reflections	49,523	47,500	52,691	35,592
No. unique reflections	12,215	10,629	11,569	10,714
Completeness (%)	92 (87)	97 (96)	98 (98)	96 (96)
Redundancy	4.1 (3.8)	4.5 (3.8)	4.6 (3.7)	3.3 (2.8)
*I*/σ(I)	13.2 (1.2)	12.4(1.7)	15(2.0)	8.3(2.0)
*R*_merge_	0.054 (0.882)	0.097 (0.698)	0.066 (0.533)	0.063 (0.686)
CC(1/2) highest resolution shell	0.72	0.83	0.77	0.7
CC(1/2)^b^ < 0.5: a^*^; b^*^; c^*^ (Å)	3.9; max; 3.7	5.6; max; 5.3	4.4; max; 5.1	4.7; max; 5.1
R_factor_(%)	24.8	28.4	25.7	25.6
R_free_^c^ (%)	28.3	30.8	28.8	30.8
rms deviations from ideal values				
Bonds (Å)	0.007	0.007	0.005	0.008
Angle (°)	1.2	1.0	1.1	1.3
Ramachandran outliers (%)^d^	0.7	0.2	0.4	0.7

a Values in parentheses refer to data in the highest resolution shell.

b this is the CC(1/2) where the resolution drops below 0.5 as reported by aimless (Evans & Murshudov, 2013).

c 5% of test reflections.

d as defined in MolProbity (Chen *et al*, 2010).

The indole moiety of L-IMH packs between the main chain of Gly219 and the side chain of Gln42 at the breaks of the bundle helices TMH6 and TMH1, respectively, and forms an edge-to-face π-stacking interaction with Trp220 (Fig1B–D). Overall, this region of the Mhp1 protein forms a hydrophobic pocket that accommodates the indole moiety of the hydantoin as shown in the surface representation in Fig1C.

To confirm unambiguously the orientation of the 5-aryl substituted ligands, the structure of Mhp1 was also solved as a complex with the bromine-containing (5Z)-5-[(3-bromophenyl)methylidene] hydantoin (BVH). Data were collected at the bromine edge, and the structure was refined at 3.7 Å (Table1). BVH binds in a very similar position and orientation to L-IMH with the bromine clearly defined in anomalous difference maps (Supplementary Fig S1C).

Having refined the IMH-Mhp1 and BVH-Mhp1 complexes, we also re-examined the BH-Mhp1 complex. Refinement was carried out against new data at a resolution of 3.8 Å (Table1). The improved electron density indicates that L-BH binds in an extended mode (Fig1E and F, and Supplementary Fig S1B) similar to L-IMH rather than the “U-shaped” folded conformation seen in the crystal structure from the small molecule database (Delgado *et al*, 2007), which was modelled previously (Weyand *et al*, 2008). The benzyl ring of L-BH overlaps the centre of the indole of L-IMH in a structural superposition within the hydrophobic binding pocket (Fig1C and E, and Supplementary Fig S1A and B).

We next examined the conformational changes that occur upon binding of ligands to Mhp1 and that can be interpreted at the modest resolution of the ligand complexes.

### Comparison of outward-open and occluded structures

A number of conformational differences within Mhp1 can be observed between the ligand-free outward-open and the ligand-bound occluded structures upon binding of all three ligands, L-IMH, L-BH and BVH (Fig1A and B). Firstly, the hash domain rotates ˜5° inwards relative to the bundle around an axis approximately parallel to TMH8 moving Trp117, Gln121 and Asn318 towards the hydantoin portion of the substrate (Fig1A and B, arrow A). In addition, the side chains of Trp117 and Trp220 swivel to sandwich the substrate in the binding site (Fig1B, arrows B and C). Gln121 in its new position is closer to and could potentially form a hydrogen bond with Gln42, whereas these two residues are 3.7 Å apart in the outward-open structure. TMH10, TMH11 and TMH12 follow the hash motif to some extent with TMH10 bending near to Ala369 to occlude the substrate in the binding site (Fig1A and B, arrow D). During these transitions, the sodium ion binding site does not appear to change appreciably.

Due to the relatively low resolution of the data for the ligand complexes and the consequent uncertainty in the exact positions of the side chains, the plausibility of the proposed hydrogen bonds to the hydantoin moiety of the ligand was tested by conducting simulations of the occluded conformation of Mhp1 with the L-BH and L-IMH substrates.

### Molecular dynamics simulations confirm the hydrogen bonding of Mhp1 to an extended configuration of the ligand

**Figure 2:**
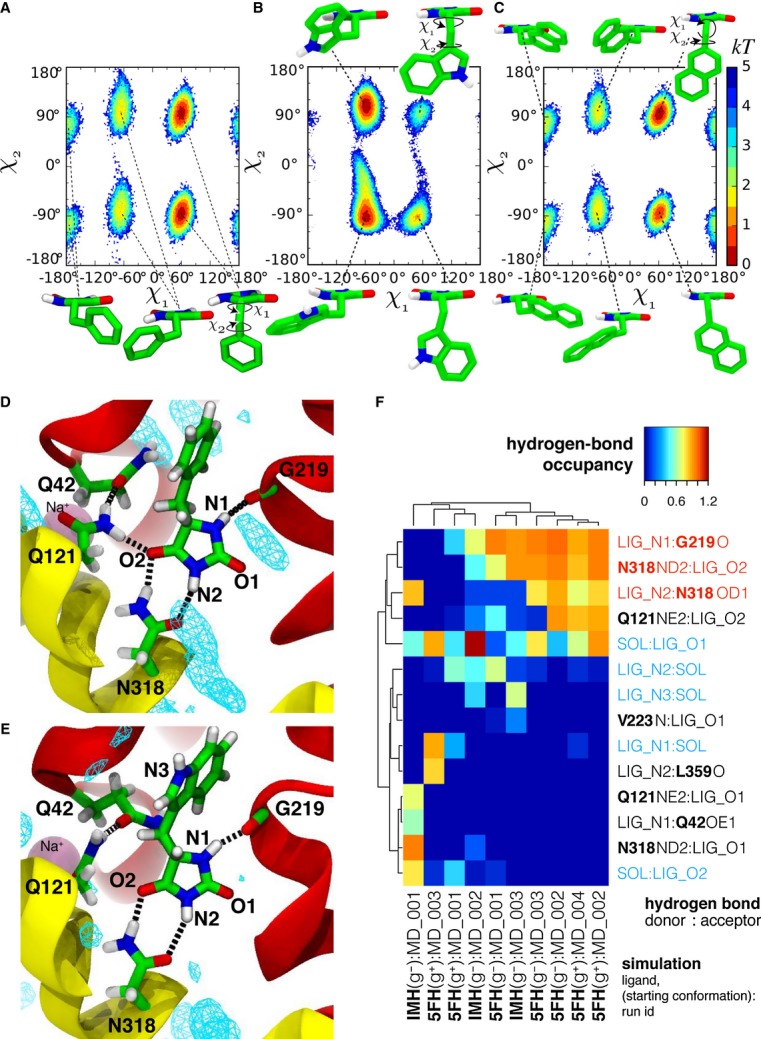
Molecular dynamics simulations of Mhp1 and its ligands A–C Molecular conformations and conformational free energy landscape of L-BH, L-IMH and L-NMH in aqueous solution suggested by molecular dynamics simulations L-BH (A), L-IMH (B) and L-NMH (C) in solution: The conformation of the hydantoin derivatives are described by the dihedral angles χ^1^ and χ^2^ as indicated in the insets. The most probable conformations are indicated by the minima in the free energy of the system (in *kT*) as a function of the dihedral angles. Regions not sampled by the equilibrium simulations are white; other possible minima would be separated by barriers larger than 6 *kT* from the accessible regions. D–F Hydrogen bonds between substrates and Mhp1 as seen in MD simulations. The ligand and important residues are shown as sticks with hydrogen bonds as broken black lines. Helices TM1 and TM6 from the bundle are in red and TM3 and TM8 from the hash motif in yellow (parts of TM3 were removed for clarity); a sodium ion in the Na2 site is visible in the background. Water density is shown as a cyan mesh, contoured at 1.5 times the bulk value. Equivalent atoms on the ligands are labelled. (D) L-BH [from simulation 5FH(*g*^+^)MD_002]. (E) L-IMH [from simulation IMH(*g*^–^)MD_002]. (F) Clustered fingerprint analysis of hydrogen bonds. The occupancy (average number of hydrogen bonds between ligand atoms and protein or solvent atoms) from all MD simulations was clustered to show the most commonly occurring hydrogen bonding patterns. Rows describe individual hydrogen bonds (identified by donor and acceptor heavy atom) while columns label individual simulations; hydrogen bonds labelled in red were also seen in the crystal structures and in docking while blue ones indicate bonds to water molecules present in the simulation. The ligand is denoted in the simulation name as either 5FH (L-BH) or IMH (L-IMH) together with the starting conformation of the χ_1_ dihedral angle and the simulation number within the set. Chemically equivalent ligand atoms are treated as the same in the analysis (indicated by the generic label “LIG” instead of “5FH” or “IMH”). Hydrogen-bonded water molecules are also treated as chemically equivalent (“SOL” for solvent).

First, we examined conformational flexibility of the substrates both in water and bound to Mhp1 using molecular dynamics simulations (see Materials and Methods and Supplementary Methods). In solution, L-BH switches freely between three major conformers, two extended forms and one U-shaped (Fig2A), as the free energy differences between them are small and the barriers are only of moderate size (< 9 *kT*) (Supplementary Fig S2). Similar results are observed for L-IMH, although the extended conformations are actually favoured (Fig2B). In contrast, when bound to Mhp1, L-BH and L-IMH become locked into an extended conformation (Supplementary Fig S3A–C, I–K), even if starting from an initial U-shaped conformation (Supplementary Fig S3E–H). The transition to the extended conformation typically occurs rapidly within the first 20 ns of the simulation.

The most frequently observed hydrogen bonds across the simulations with the Mhp1 ligand-occluded form are: a two-pronged interaction between one N/O face of the hydantoin ring with Asn318; between N1 of the hydantoin and the backbone of Gly219; and partially between O4 of the hydantoin and Gln121 (Fig2D and E). The remaining oxygen in the hydantoin moiety tends to be solvated by water, with the region near TMH8 being the only fully solvated part of the binding site (Fig2D and E). On the limited time scale of the simulations, hydrogen bonds to Gln42 were not observed with the crystallographically observed conformer. In a single simulation, the hydantoin moiety rotated by 180° and adopted an alternative binding mode that included a transient hydrogen bond between the hydantoin and Gln42 instead of the persistent bond to Gly219 [IMH(g-):MD_001 in Fig2F and Supplementary Fig S3i]. Across the simulations, a common pattern emerges (Fig2D–F and Supplementary Fig S3) whereby an extended conformation of the ligand is required so that the hydantoin ring can hydrogen bond simultaneously to Asn318 and Gly219. Overall, the simulations corroborate the existence of the proposed H-bonds to Asn318, Gly219 and possibly Gln121.

After identifying the overall conformational changes of Mhp1 upon binding of ligand, we sought to establish the roles of individual residues in ligand binding using a site-directed mutagenesis approach.

### The roles of individual residues of Mhp1 in ligand binding and transport

The effect of mutating individual residues, especially those suggested to interact with the ligands in the above structures and simulations, was investigated. Changes were monitored in the uptake of radioisotope-labelled substrates into cells and in the direct binding of ligands to purified protein, measured using spectrophotofluorimetry (Materials and Methods).

**Figure 3:**
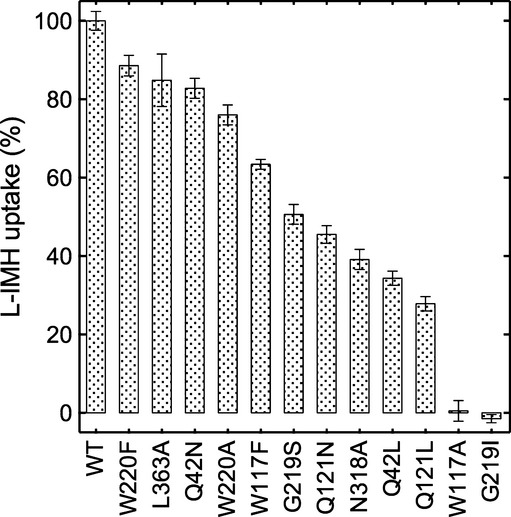
Impairment of hydantoin uptake in mutants of Mhp1 The accumulation of ^14^C-L-IMH (50 μM initial external concentration) was measured for 15 s in cells expressing the wild-type or mutant Mhp1 proteins (Materials and Methods and Supplementary Methods). All measurements were normalised to percentages by comparison with the wild-type value of 0.57 ± 0.01 (s.e.m., *n* = 34) nmol/mg dry mass. Error bars represent the s.e.m. of at least triplicate measurements. All assays were performed in the presence of 150 mM NaCl.

Replacement of the completely conserved Asn318 with an alanine led to a significant loss of uptake activity (Fig3 and Supplementary Table S1) and a substantial reduction in binding (Supplementary Table S1 and Supplementary Fig S4) as might be expected from the loss of the bidentate hydrogen bonding arrangement seen in the structure (Fig1). The conservative mutation of Gln121 to asparagine resulted in partial decreases in efficiency of both uptake and binding (Fig3, Supplementary Table S1 and Supplementary Fig S4), while its replacement with leucine, as is observed in the uridine transporter Fui1 (de Koning & Diallinas, 2000; Weyand *et al*, 2008), reduced uptake and binding yet further. This is again consistent with the hydrogen bonding interaction proposed from the structures and simulations (Figs1 and 2).

Gly219 of Mhp1 is not well conserved amongst NCS1 family members, but in Mhp1, it is a key component of the break in TMH6 that contributes to accommodation of the ligand; in addition, its carbonyl oxygen forms a hydrogen bond with the L-IMH. Substitution of this residue with serine or isoleucine as seen in other NCS1 transporters reduced both binding and transport activity (Fig3, Supplementary Table S1 and Supplementary Fig S4), with the more bulky isoleucine having a more pronounced effect as would be expected. In the IMH-Mhp1 crystal structure, there is very limited space available in the region of Gly219 such that substitution of this glycine with any other amino acid would result in a clash of the amino acid side chain with the indole ring of L-IMH.

Trp117 and Trp220 sandwich L-IMH in the binding pocket. The aromatic ring of Trp117 seems to be important for uptake because activity is reduced dramatically if this residue is replaced by an alanine but only moderately when changed to a phenylalanine (Fig3 and Supplementary Table S1). Surprisingly, the change of Trp220, either conservatively to phenylalanine, or more drastically to alanine, had little effect on uptake (Fig3 and Supplementary Table S1), despite the conservation of this residue in aligned NCS1 transporters.

From measurements of uptake and binding activities with the Gln42Asn and Gln42Leu mutants (3Fig and Supplementary Table S1), this residue would appear to play an important role in the mechanism of Mhp1, but the basis for this is not obvious from the crystal structure or molecular dynamics simulations. Gln42 is within van der Waals interaction distance of the aromatic rings of L-IMH and L-BH and in fact was difficult to position in the crystal structure due to the close interaction between the protein and ligand atoms. It is not within hydrogen bonding distance of any atom from L-IMH, although the side chain can potentially interact with the π electrons of the indole ring. Instead, it forms a hydrogen bonding interaction with Gln121 (Fig2E). Shortening the side chain by mutating Gln42 to asparagine resulted in modest decreases in uptake and binding efficiency, while the respective reductions were greater when Gln42 was replaced with phenylalanine or leucine (Fig3, Supplementary Table S1 and Supplementary Fig S4). While the replacement with a bulky hydrophobic group presumably causes steric hindrance preventing the substrate from binding, mutation to asparagine could result in a reduction of affinity either as a direct result of the loss of the interaction with the ligand or a disruption of the hydrogen bonding interaction with Gln121. As neither Gln121 nor Gln42 are conserved amongst the wider NCS1 family, it seems unlikely that this latter interaction is instrumental in inducing the conformational changes necessary for switching the transporter from outward to inward facing, or indeed other conformational changes, such as inward to outward facing. A possible role for Gln42 is in shaping the binding pocket to enable the substrates to bind although we cannot exclude that it affects the binding of the nearby sodium ion.

The above crystal structures, simulations and mutagenesis studies strongly suggested that the hydrogen bonding network with the hydantoin moiety is critical for binding. We then sought to expand our understanding of the interactions between the protein and its substrates using molecular modelling and synthetic chemistry to generate new ligands for Mhp1.

### The structure–activity relationship of ligands binding to Mhp1

**Figure 4:**
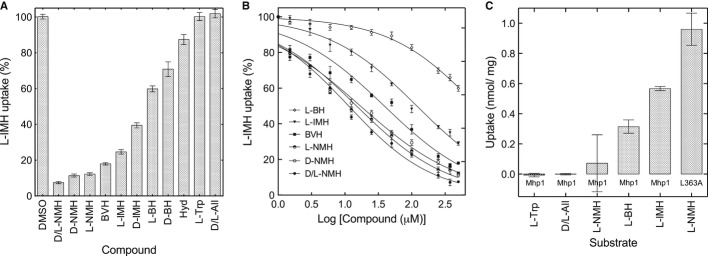
Ligand specificity of Mhp1 determined by uptake assays A, B Accumulation of ^14^C-L-IMH (50 μM initial external concentration) into wild-type cells was measured for 15 s (Materials and Methods and Supplementary Methods): (A) in the presence of 500 μM of the indicated unlabelled compound; and (B) dose–response data for ^14^C-L-IMH uptake in the presence of 0–500 μM of selected unlabelled compound. All measurements were normalised to percentages by comparison with the wild-type value of 0.57 ± 0.01 (s.e.m., *n* = 34) nmol/mg dry mass, and the error bars represent the s.e.m. of at least triplicate measurements. C Uptakes of the indicated radioisotope-labelled compounds (50 μM initial external concentration, Materials and Methods and Supplementary Methods), into wild-type cells were measured for 15 s for the original Mhp1 protein and for the L363A mutant as indicated at least in triplicate on each of two cell preparations and the s.e.m. calculated for at least six assays. Hyd = hydantoin; All = allantoin. L-tryptophan and D/L allantoin were tested as controls in both (A) and (C).

To investigate the structure–activity relationship of potential ligands, we tested their binding to Mhp1 by measuring inhibition of ^14^C-L-IMH uptake into whole cells. We first tested known substrates of the related NCS1 transport proteins, including allantoin, adenosine, uracil, guanosine, cytosine, thiamine and nicotinamide riboside (Supplementary Fig S5). Allantoin contains a hydantoin moiety but, surprisingly, did not show any inhibition of transport activity by Mhp1 (Fig4A). All of the other compounds, which do not have this moiety, produced very weak or no inhibition (Supplementary Fig S5). Hydantoin was found to reduce the uptake of the radio-labelled IMH (Fig4A, Table2 and Supplementary Fig S5), much less than either L-BH or L-IMH, implying that the substituent in position 5 plays an important role. Thus, apparently the hydantoin moiety is required, but is not sufficient on its own for effective binding to Mhp1.

**Table 2:** Impairment of ^1^^4^C-L-IMH uptake by selected ligands and their binding by Mhp1

Compound	Structure	Residual uptake (%)	Apparent IC_5__0_ (μM)	Apparent *K*_*d*_ (μM)
D-NMH	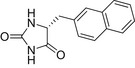	11.4 ± 0.9	17.1 ± 1.1	1.8 ± 0.3
L-NMH	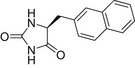	12.3 ± 0.8	14.9 ± 1.1	3.6 ± 0.8
BVH	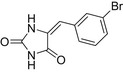	18.0 ± 0.7	38.8 ± 1.1	2.0 ± 0.2
L-IMH	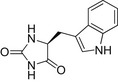	24.7 ± 1.3	119.2 ± 1.1	19.0 ± 2.0
D-IMH	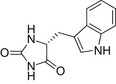	39.9 ± 1.5	ND	20.6 ± 3.8
L-BH	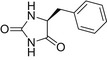	59.9 ± 1.7	945.0 ± 1.1	36.0 ± 1.4
D-BH	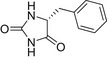	70.9 ± 4.1	ND	130.0 ± 10.0
Hydantoin	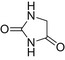	87.4 ± 2.8	ND	NC
L-Tryptophan	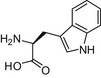	100.3 ± 2.2	ND	ND
D/L-Allantoin	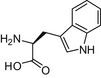	101.9 ± 2.3	ND	NC

Percentage uptake and apparent IC_5__0_ values were determined by a ^1^^4^C-L-IMH ligand uptake assay, and apparent *K*_*d*_ values were determined by stopped-flow spectrophotofluorimetry. All measurements were taken in the presence of 150 mM NaCl (see Materials and Methods and Supplementary Methods) and are shown with the associated standard errors of the mean. NC denotes “not converged” indicating that an apparent *K*_*d*_ or IC_5__0_ value could not be determined by fitting to a rectangular hyperbola. ND denotes “not determined”.

**Figure 5:**
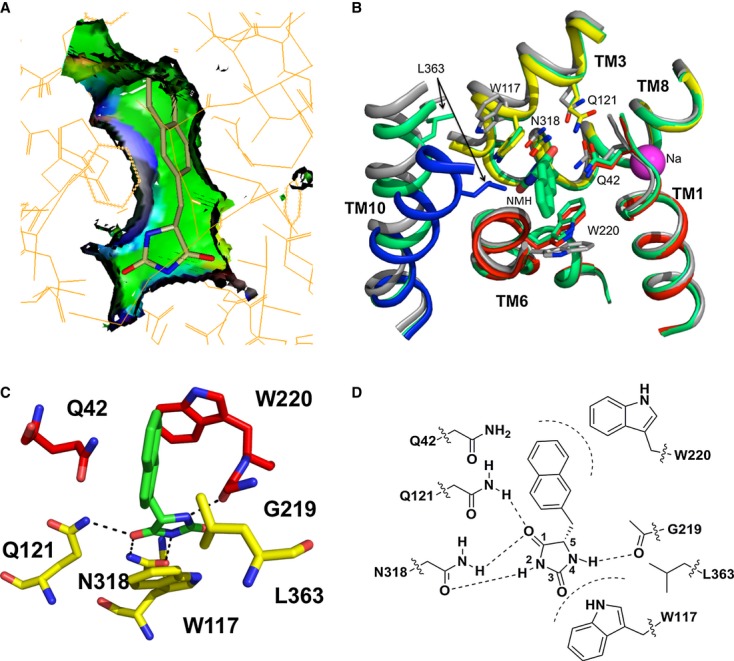
Structure of wild-type Mhp1 with bound L-NMH A Docking pose of L-NMH illustrated using SPROUT as for Fig1. B Comparison of the crystal structure of L-NMH (green) with the outward-open ligand-free structure (grey) and the complex with L-IMH (coloured as in Fig1). C, D Potential hydrogen bonding interactions between L-NMH and the protein as in Fig1.

Next, we explored the structure–activity relationship of the 5-substituent moiety of the ligand with the choice of compounds guided by docking algorithms (Materials and Methods; Supplementary Methods). The crystal structures showed that the indolylmethyl or benzyl group of the original substrates binds in a large hydrophobic pocket bounded primarily by residues Ile45, Phe216, Gly219 and Trp220 (Fig1). Docking studies suggested that the naphthyl moiety of NMH would fit into the hydrophobic pocket (Fig5A). In fact, this molecule displayed the most effective inhibition of ^14^C-L-IMH uptake of all compounds tested (Fig4A and B, Table2 and Supplementary Fig S5). Overall, both the inhibitions of uptake and the apparent affinities of Mhp1 for selected ligands, measured using fluorimetry, decreased in the order NMH > BVH > IMH > BH (Fig4A and B, Table2 and Supplementary Fig S5). These assays also showed that Mhp1 generally binds the L-enantiomers of 5-substituted hydantoins with higher affinity than the respective D-enantiomer (Table2, Fig4A, and Supplementary Fig S5) (Suzuki & Henderson, 2006). For NMH, however, the two enantiomers bind with affinities that are indistinguishable within the experimental error (Fig4A and B, Table2 and Supplementary Fig S5).

Having identified NMH as the compound with the tightest binding to Mhp1 as measured in the inhibition and binding assays (summarised in Table2), we sought to establish the details of its interaction with the protein using crystallography.

### Crystal structure of Mhp1 with bound NMH

Mhp1 was cocrystallised with racemic NMH, and the structure of the resulting complex was refined at 3.7 Å (Table1). Electron density was observed in the ligand binding site, consistent with the extended forms of both the L- and D-enantiomers of NMH (Supplementary Figs S1D and S7). The hydantoin moieties of both enantiomers make similar interactions to those seen for L-IMH and L-BH (Fig5B–D), and the naphthyl groups of each enantiomer substantially occupy the hydrophobic pocket between TMHs 1 and 6, even more so than the benzyl and indolylmethyl groups of L-BH and L-IMH, which is consistent with the predictions from the docking studies (Figs1C and E and 5A).

Upon binding NMH, the protein undergoes similar conformational changes to those described above for the L-IMH-bound state with a movement of the hash domain relative to the bundle and a rotation of the two tryptophans (Fig5B). Rather surprisingly, however, TMH10 remains in the open position. If TMH10 were to adopt the same conformation that is observed in the complex with IMH, then Leu363 may clash with the naphthyl ring of NMH (3 Å distance in the low-resolution crystal structure) (Fig5B). Thus, the bulky naphthyl substituent appears to interfere with the formation of the occluded conformation of TMH10.

### NMH is an inhibitor and not a substrate for Mhp1

The observation that TMH10 was in the open position in the complex with NMH suggested that NMH may act as an inhibitor rather than a substrate. Were this to be the case, it was hypothesised that removing the proposed steric clash at Leu363 (Fig5B) by mutating the protein could restore transport of NMH. This was investigated by synthesising radio-labelled L-NMH and comparing its uptake with those for other radio-labelled substrates (see Materials and Methods, Supplementary Methods and Fig4C).

Consistent with its action as an inhibitor rather than a substrate no significant uptake of L-NMH into wild-type cells was observed (Fig4C), despite this compound inhibiting L-IMH uptake very substantially (Fig4A and B*,* Table2). In contrast, when Leu363 was mutated to an alanine, uptake was restored (Fig4C), substantiating our hypothesis, based on the crystal structure, that TMH10 must occupy a defined closed position for transport to occur.

## Discussion

Here, we have determined the structure of Mhp1 with four different 5-substituted hydantoin derivatives, L-IMH, L-BH, BVH and D/L-NMH. The combination of improved maps due to higher-resolution data along with anomalous difference maps derived from the bromo-substituted compound BVH has allowed us to assign unambiguously the position and orientation of the ligands. Furthermore, the existence and nature of hydrogen bonds stabilising the hydantoin moiety and the importance of a hydrophobic pocket accommodating an extended conformation of a 5-substituent have been substantiated by a combination of mutagenesis, molecular dynamics simulations and comparison of binding efficiencies of different ligands. Ligands with a hydantoin moiety bind with higher affinity than those with other nucleobase-like entities. This specificity is conferred by hydrogen bonding interactions with Asn318 and Gln121, which are conserved residues on the hash motif, and with the carbonyl oxygen of Gly219 located at the breakpoint of TMH6 on the bundle. In addition, the conserved Trp117 residue of the hash motif forms an important π-stacking interaction with the hydantoin moiety. Similar results were also obtained for other NCS1 members, the eukaryotic purine–cytosine transporter, FcyB from *Aspergillus nidulans* (Krypotou *et al*, 2012) and the plastidic nucleobase transporter from *Arabidopsis thaliana*, PLUTO (Witz *et al*, 2014). In these studies, which were based on the structure of Mhp1, the equivalent residues to Trp117, Asn318 and Gln121 were all shown to be important for substrate binding, although the magnitude of the effect varied amongst the proteins. The specificity for the 5-substituent appears to be less strict because a range of hydantoin derivatives can bind to Mhp1. Nevertheless, the clear preference for larger, more extended hydrophobic and aromatic moieties can be explained by the hydrophobic pocket located predominantly within the bundle region of the protein, between TMHs 1 and 6. Most importantly, both the hydantoin and 5-substituent groups are necessary for tight binding and uptake.

**Figure 6:**
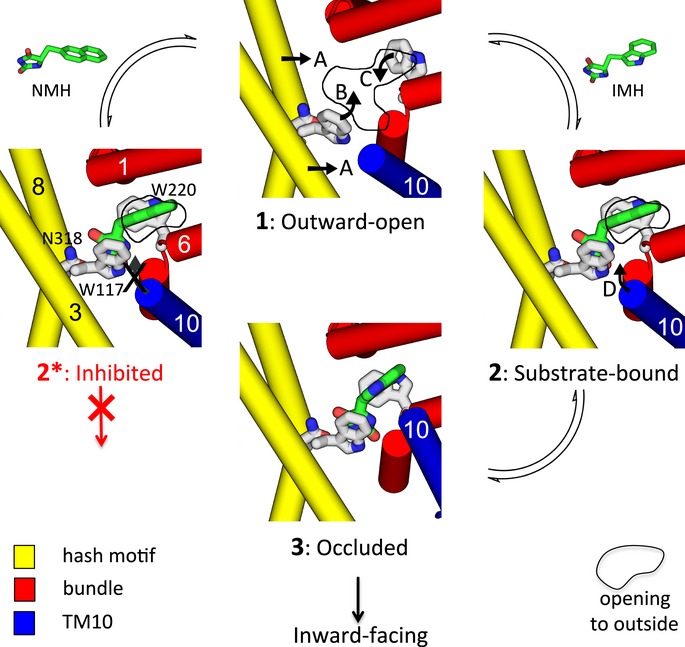
Scheme for binding of ligands and transport by the Mhp1 protein Upon binding to the outward-open conformation of Mhp1 (1) both the substrate IMH (right) and the inhibitor NMH (left) induce a number of conformational changes to the protein. The hash motif (yellow) moves towards the bundle (red), and Trp117 and Trp220 rotate to interact with the ligand (denoted by arrows A, B and C respectively). This results in a partial occlusion of the outward cavity, shown here by a solid line approximately defining the entrance to the cavity from the outside. A conformational change of TMH10 (D) results in the complete occlusion of the substrate in the binding site (3), and subsequently, the protein switches to the inward-facing form. For NMH (2*), TMH10 cannot adopt the position observed for NMH and transport does not occur. The scheme has been based on the crystal structures of states 1, 2* and 3.

Sodium ions binding at the interface between the hash motif and the bundle have been postulated to favour the formation of the outward-facing state (Weyand *et al*, 2011), as has been measured by single molecule FRET for LeuT (Zhao *et al*, 2011) and conjectured for other superfamily members (Abramson & Wright, 2009; Krishnamurthy *et al*, 2009; Perez & Ziegler, 2013). In this conformation, the protein would be ready to accept the substrate. In the sodium-bound outward-open form of Mhp1, there is a clear cavity for the substrate to enter and bind although the residues involved in binding are not in optimal positions to accommodate the ligand. Instead, the substrate induces a number of conformational changes in the protein. Firstly, there is a rigid body rotation of the hash domain relative to the bundle bringing the conserved Trp117 and Asn318 closer to the substrate (Fig6). Secondly, Trp117 and Trp220 each rotate slightly to pack onto the hydantoin and hydrophobic moieties of the ligand, respectively (Fig6). These changes have consistently been observed in all four Mhp1–ligand complexes presented here. The next conformational change is caused by the packing of TMH10 onto the substrate (Fig6). Although this change was reported previously, we are now in a position to discuss its significance in more depth.

In the outward-open ligand-free structure, TMH10 is relatively straight, but when substrate binds, it bends over the substrate, occluding it in the binding site (Fig1A). Molecular dynamics simulations have suggested that when the protein is outward facing, this helix is in equilibrium between open and closed states (Shimamura *et al*, 2010; Adelman *et al*, 2011; Shi, 2013). The crystallographic data reported here are in agreement with these studies but furthermore suggest that TMH10 switches between discrete favourable states. When the putative natural substrates, that is*,* L-IMH or L-BH are bound, TMH10 is in the closed position (Fig6); however, when the slightly bulkier NMH is present in the binding site, the helix retains the conformation seen in the ligand-free structures (Fig6). The results from radioactive transport assays demonstrate that the Leu363Ala mutation changes NMH from an inhibitor into a substrate. This suggests that a steric clash between Leu363 and the bulky naphthyl group of NMH (Figs5B and 6) prevents this compound from being transported. Hence, only when TMH10 is completely closed can transport be effected. Thus, it can be conjectured that once Na^+^ and substrate have bound, it is the closure of the thin gates that triggers the transition to the inward-facing conformation. Presumably, although TMH10 is mobile when in the outward-open ligand-free state, it cannot adopt the required conformation necessary for transition to the inward-facing conformation if the other conformational changes that accompany substrate binding do not occur. At the resolution of the crystal structures, the sodium ion is not clearly defined and it is difficult to discuss further how the sodium ion binding site is affected by the presence of the substrate.

As more structures are solved of members of the 5HIRT superfamily, we are accumulating more information about the similarities and differences in their mechanisms of transport (Shimamura *et al*, 2010; Krishnamurthy & Gouaux, 2012; Perez *et al*, 2012; Shi, 2013). Although the location of the substrate-binding site between the bundle and hash motif is similar in all of these structures, the exact binding mode of the diverse substrates varies. While we show here that the substrates form hydrogen bonding interactions mainly with the hash motif in Mhp1, in the other structures, most specific interactions are observed with the bundle motif (Yamashita *et al*, 2005; Faham *et al*, 2008; Fang *et al*, 2009; Gao *et al*, 2009; Ressl *et al*, 2009; Shaffer *et al*, 2009; Schulze *et al*, 2010; Tang *et al*, 2010; Kowalczyk *et al*, 2011; Ma *et al*, 2012; Perez *et al*, 2012). It can be speculated that this is one of the reasons for the more rigid movement of the two domains with respect to one another in Mhp1 than is so far apparent for other members of the 5HIRT family (Shimamura *et al*, 2010; Krishnamurthy & Gouaux, 2012; Perez *et al*, 2012). In going from outward-open to occluded, the sodium symporters LeuT and BetP and the antiporter AdiC all show a movement of the hash motif relative to the bundle as observed for Mhp1. However, in these proteins, there is more flexing of the helices of the bundle domain around the breakpoints of TMH1 and TMH6 where the relative substrates bind (Gao *et al*, 2010; Krishnamurthy & Gouaux, 2012; Perez *et al*, 2012). The competitive inhibitor tryptophan holds LeuT in the outward-facing conformation preventing this transition (Singh *et al*, 2008). As we have performed for Mhp1 with NMH, a simple mutation in LeuT can convert tryptophan into a substrate (Piscitelli & Gouaux, 2012) although this mutation occurs on TMH8 rather than TMH10. Indeed, as might be expected for different substrates, the occlusion mechanism varies from one transporter to another and only Mhp1 shows such a dramatic movement of TMH10. In BetP and AdiC, the movement is much more subdued (Gao *et al*, 2010; Perez *et al*, 2012), and in LeuT, there is very little difference in its position amongst the various crystal structures (Krishnamurthy & Gouaux, 2012). In fact, amongst all the solved structures of the 5HIRT superfamily, the conformation of TMH10 seen in the occluded and inward-facing forms of Mhp1 is only observed in the inward-facing vSGLT (Faham *et al*, 2008). Thus, although the core fold is similar amongst these proteins, the details of substrate recognition and the conformational changes that occur upon substrate binding differ. This study, combined with recent observations on other members of the family, is revealing how transporters of the 5HIRT family evolved to recognise different substrates (and cations) and implement symport, antiport or uniport functions while retaining underlying similarities in protein fold and molecular mechanism of translocation.

In summary, by combining crystallography with molecular dynamics, genetic manipulation, biochemical/biophysical assays, and, importantly, computational and synthetic chemistry, we have been able to analyse the exquisite precision by which Mhp1 recognises substrates and discover more potent ligands. Furthermore, we have described a novel intermediate conformation of the protein and shown that transport cannot be effected without closure of the external thin gate. These insights will expand further our understanding of the effectiveness of known antimycotic (Paluszynski *et al*, 2006; Chen *et al*, 2011) and antibacterial (Imperi *et al*, 2013) drugs as well as promote the development of novel microbial pathways for syntheses of chiral compounds (Bommarius *et al*, 1998; Altenbuchner *et al*, 2001; Suzuki *et al*, 2005; Matcher *et al*, 2012).

## Materials and Methods

### Cell growth and expression of the Mhp1 protein

For subsequent purification of Mhp1 protein for fluorescence measurements, cells of *Escherichia coli* BL21(DE3) transformed with plasmid pSHP11 encoding the *hyuP* gene from *Microbacterium liquefaciens* AJ3912 were cultivated and induced for expression of Mhp1 as described previously (Suzuki & Henderson, 2006) but in larger scale 30 or 100 litre fermenters. Details are described in Supplementary Methods. Expression and purification of Mhp1 for cocrystallisation with ligands is described in detail in Supplementary Methods. For small-scale growth of the same cells for subsequent measurements of uptake of radioisotope-labelled compounds, a variation on the procedure for growth, induction, harvesting, washing and resuspension was adopted, and the details are described in Supplementary Methods.

### Assays of uptake of radioisotope-labelled compounds

^14^C-labelled compound, generally L-IMH (Patching, 2011), (50 μM final concentration) was added to the cells in 150 mM NaCl, 5 mM MES pH 6.6, and the appearance of radioactivity in the cells was measured after 15 s and 2 min. For competition assays, unlabelled compound (500 μM) was added 3 min beforehand. The mean radioactivity was converted to nmol/mg dry mass at each time point and expressed as percentage of the controls without unlabelled compound. In selected cases dose–response curves were obtained, which allowed apparent IC_50_ values to be generated using Graph-Pad Prism 6 software.

### Synthesis of selected ligands

Synthesis of D/L-NMH, BVH, D/L-IMH, D/L-BH, para-methyl-D/L-BH, para-ethyl-D/L-BH and para-propyl-DL-BH followed a simple 1- or 2-step procedure (Supplementary Figs S8 and S9). Condensation of the appropriate aldehyde with hydantoin followed by hydrogenation of the alkene moiety gave the desired 5-substituted hydantoin as a racemic mixture. The geometry of the synthesised alkenes was determined to be Z by reference to published NMR studies (Thenmozhiyal *et al*, 2004).

The enantiomerically pure hydantoin derivatives D-NMH, L-NMH, L-IMH, D-IMH, p-methyl-L-BH, p-methyl-D-BH, L-BH and D-BH were synthesised by condensation of the appropriate α-amino acid with potassium cyanate via the N-carbamoyl-α-amino acid (Supplementary Methods). A ^14^C-labelled version of L-NMH was synthesised by including [^14^C]potassium cyanate in the reaction mixture (Supplementary Methods).

### Protein crystallisation and structure determination

Crystals of IMH-Mhp1, BH-Mhp1, BVH-Mhp1, NMH-Mhp1 were grown essentially as previously described (Shimamura *et al*, 2008, 2010; Weyand *et al*, 2008). Details of crystallisation, data collection and structure refinement are in Supplementary Methods.

### Determination of dissociation constant, *K*_*d*_, for binding of ligands to Mhp1

In principle kinetic constants for binding of ligands to Mhp1 can be determined by measuring changes in fluorescence (ΔF) of its tryptophan residues in response to titration of the protein with a test ligand in a suitable buffer (10 mM Tris pH 7.6, 0.05% DDM, 2% DMSO, 15 mM NaCl and 125 mM choline chloride with 140 μg/ml Mhp1 at 18°C) under steady-state conditions (Weyand *et al*, 2008). In practice, in order to overcome interference by absorption of the ligand itself, titrations were performed and ΔF was measured by a stopped-flow non-equilibrium method, details of which are given in Supplementary Methods with example binding curves shown in Supplementary Fig S6. In the case of mutations in Trp117 and Trp220, as both are likely to contribute to the fluorescence change seen when ligands bind, measurement of transport is a more reliable indicator of their roles in function than fluorimetric measurements of ligand binding.

### Molecular dynamics simulations

The ligands L-BH, L-IMH and L-NMH were parameterised with the OPLS-AA force field (Rizzo & Jorgensen, 1999) and the MOL2FF algorithm (Beckstein & Iorga, unpublished). Equilibrium MD simulations of each ligand molecule in water were performed at *T *=* *300 K and *P *=* *1 bar with Gromacs 4.5.3 (Hess *et al*, 2008) for 100 ns (L-BH, L-NMH) or 200–500 ns (L-IMH). The free energy landscape in the dihedral angles was created with the MDAnalysis tool kit (Michaud-Agrawal *et al*, 2011) as described in Supplementary Methods. MD simulations of conformations of the Mhp1 protein with L-BH or L-IMH bound followed our previous work (Shimamura *et al*, 2010) with some differences as described in Supplementary Methods. Briefly, the OPLS-AA force field (Rizzo & Jorgensen, 1999) was used for protein, ligands and POPC lipids. The system was simulated at constant temperature (310 K) and pressure (1 bar) and a free NaCl concentration of ˜100 mM. Multiple simulations were carried out with different initial conformations of the ligand. For analysis, hydrogen bonds were detected by a geometric criterion with hydrogen-acceptor distance ≤ 3.5 Å and the bond angle < 30°.

### Design and selection of novel ligands for Mhp1

Compounds were designed using the SPROUT *de novo* design program (Law *et al*, 2003). The original crystal structure of the ligand-bound Mhp1 complex (PDB code: 2JLO) was used as the basis for new templates. New templates were designed using the Maestro molecular modelling environment (http://www.schrodinger.com/productpage/14/12/). These were then docked into the Mhp1 crystal structure using the electronic high-throughput screening programme eHiTS (Zsoldos *et al*, 2006), which utilises an exhaustive systematic search algorithm that considers all docking poses that lack severe steric clashes with the protein. Compounds were chosen for synthesis based upon their predicted binding affinity to the protein as determined using the eHiTS and SPROUT scoring functions and also their predicted binding pose, as visualised using the SPROUT protein boundary surface representation.

### Accession codes

Protein Data Bank: the newly acquired crystal structures for IMH-Mhp1, BH-Mhp1, BVH-Mhp1 and NMH-Mhp1 are deposited under access codes 4d1a, 4d1b, 4d1c and 4d1d, respectively.

## Abbreviations

Mhp1: Microbacterium hydantoin permease 1
BH: 5-benzyl-L-hydantoin = (5S)-5-benzylimidazolidine-2,4-dione
BVH: 5-bromovinylhydantoin = (5E)-5-[(3-bromophenyl)methylidene] imidazolidine-2,4-dione
IMH: 5-indolylmethyl-L-hydantoin = (5S)-5-(1H-indol-3-ylmethyl)imidazolidine-2,4-dione
NMH: 5-(2-naphthylmethyl)-L-hydantoin = (5S)-5-(naphthalen-2-ylmethyl)imidazolidine-2,4-dione
All: allantoin = 5-ureidohydantoin = (2,5-dioxo-4-imidazolidinyl) urea
Hyd: hydantoin = imidazolidine-2,4-dione
