# Supplementary material for: Molecular mechanism of ligand recognition by membrane transport protein, Mhp1
Source: EMBO J. 2014 Jun 21;33(16):1831–44. doi: 10.15252/embj.201387557 (PMC4195764; doi:10.15252/embj.201387557)
Supplement: Supplementary file 1 [file embj0033-1831-sd1.pdf]

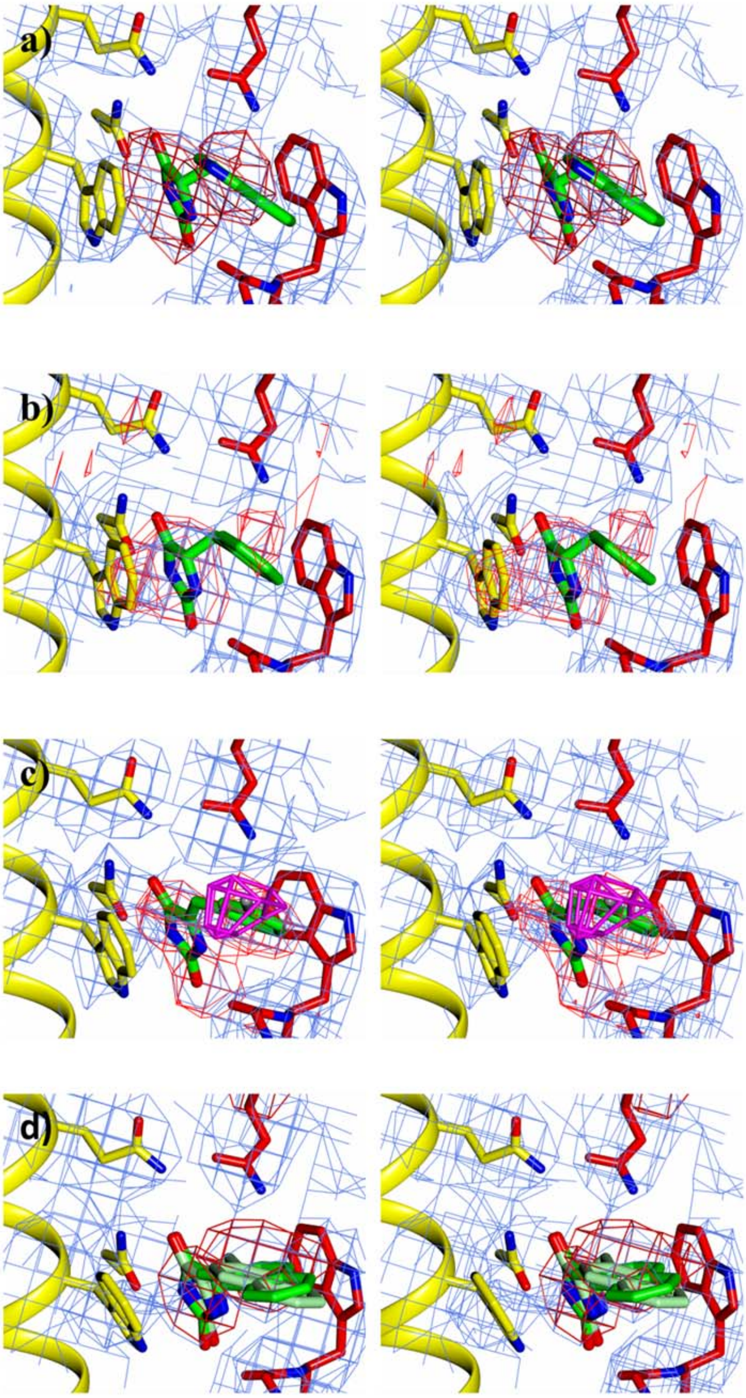

Figure S1. Electron density for the hydantoin derivatives in the binding site of Mhp1. (**a**) Complex with L-IMH. The side chains are coloured as in **Figure 1**. The associated electron density was calculated using phases derived from the model before the ligand was added. The B-factor sharpened 2mFo-DFc density is shown in blue (1σ) and the mFo-DFc in red (3.5σ). (**b**) As (**a**) for L-BH. The density maps are contoured at 1.5 and 2.3σ respectively for the 2mFo-DFc and mFo-DFc. (**c**) As (**a**) but for BVH and with an anomalous difference map contoured at 8.5σ shown in purple. Good correspondence is seen with the bromine atom. The 2mFo-DFc and mFo-DFc maps are contoured at 1.8 and 3.6σ respectively. (**d**) As (**a**) for NMH. The L and D forms of the compound are shown in green and light-green respectively. The density maps are contoured at 1.5 and 3σ respectively for the 2mFo-DFc and mFo-DFc maps.
