# Supplementary material for: Molecular mechanism of ligand recognition by membrane transport protein, Mhp1
Source: EMBO J. 2014 Jun 21;33(16):1831–44. doi: 10.15252/embj.201387557 (PMC4195764; doi:10.15252/embj.201387557)
Supplement: Supplementary file 2 [file embj0033-1831-sd2.pdf]

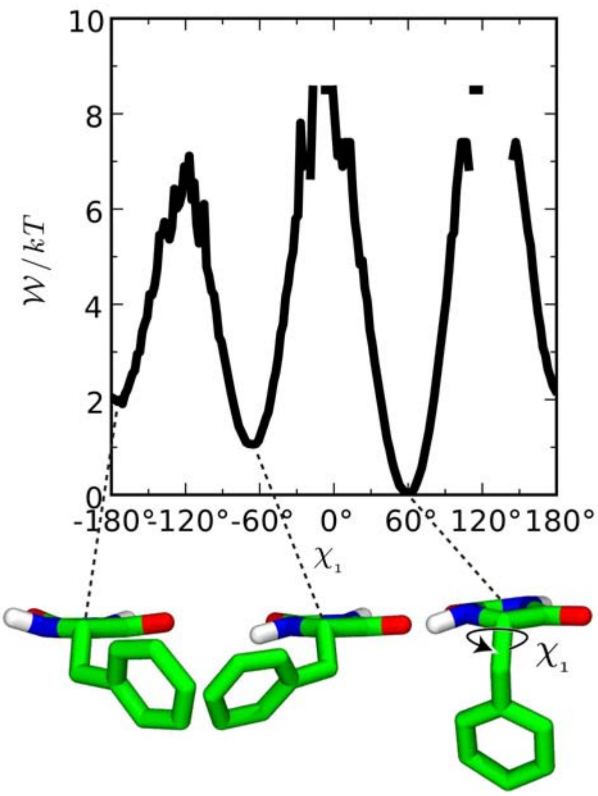

Figure S2. Free energy landscape of L-BH as a function of dihedral angle χ_1 alone. Due to the symmetry of the phenyl ring, the major conformations of L-BH are described by only one torsion χ_1 and the resulting free energy landscape displays characteristic minima for the *anti* (–180º), g^- (–60º) and g^+ (+60º) conformer. As the 100-ns equilibrium MD simulation of L-BH in solution contained multiple transitions between these minima (especially *anti* ↔ g^-↔ g^+, **Supplementary Fig. 3d**), the barrier for interconversion between conformers is estimated to be < 9 *kT* (between g^- and g^+, the barrier between g^+ and *anti* is higher but interconversion can proceed through the pathway with the lower barrier).
