# Supplementary material for: Molecular mechanism of ligand recognition by membrane transport protein, Mhp1
Source: EMBO J. 2014 Jun 21;33(16):1831–44. doi: 10.15252/embj.201387557 (PMC4195764; doi:10.15252/embj.201387557)
Supplement: Supplementary file 3 [file embj0033-1831-sd3.pdf]

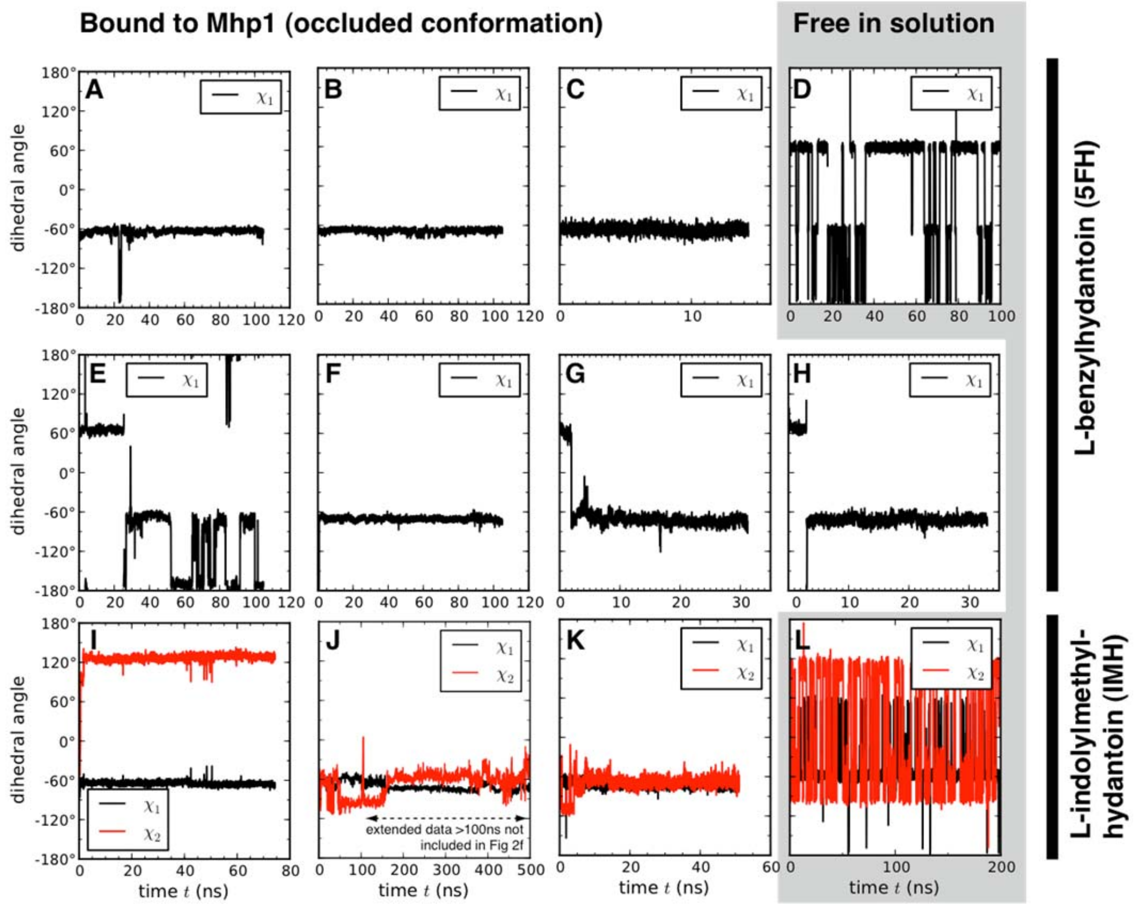

Figure S3. Time evolution of dihedral angles in L-BH (ligand code 5FH) and L-IMH from MD simulations. (**A-C**) Simulations 5FH(g^{-}) MD_001–MD_003 (L-BH bound to Mhp1, started from the extended conformation as seen for the IMH-bound structure). (**D**) L-BH in solution. (**E-H**) Simulations 5FH(g^{+}) MD_001–MD_004 (L-BH bound to Mhp1, started from the U-shaped folded over conformation as modeled in 2JLO). (**I-K**) Simulations IMH(g^{-}) MD_001–MD_003 (L-IMH bound to Mhp1 as seen in the crystal structure). (**I**) L-IMH in solution. (**J**) Extended to 500 ns. (**L**) L-NMH in solution.
