# Supplementary material for: Molecular mechanism of ligand recognition by membrane transport protein, Mhp1
Source: EMBO J. 2014 Jun 21;33(16):1831–44. doi: 10.15252/embj.201387557 (PMC4195764; doi:10.15252/embj.201387557)
Supplement: Supplementary file 4 [file embj0033-1831-sd4.pdf]

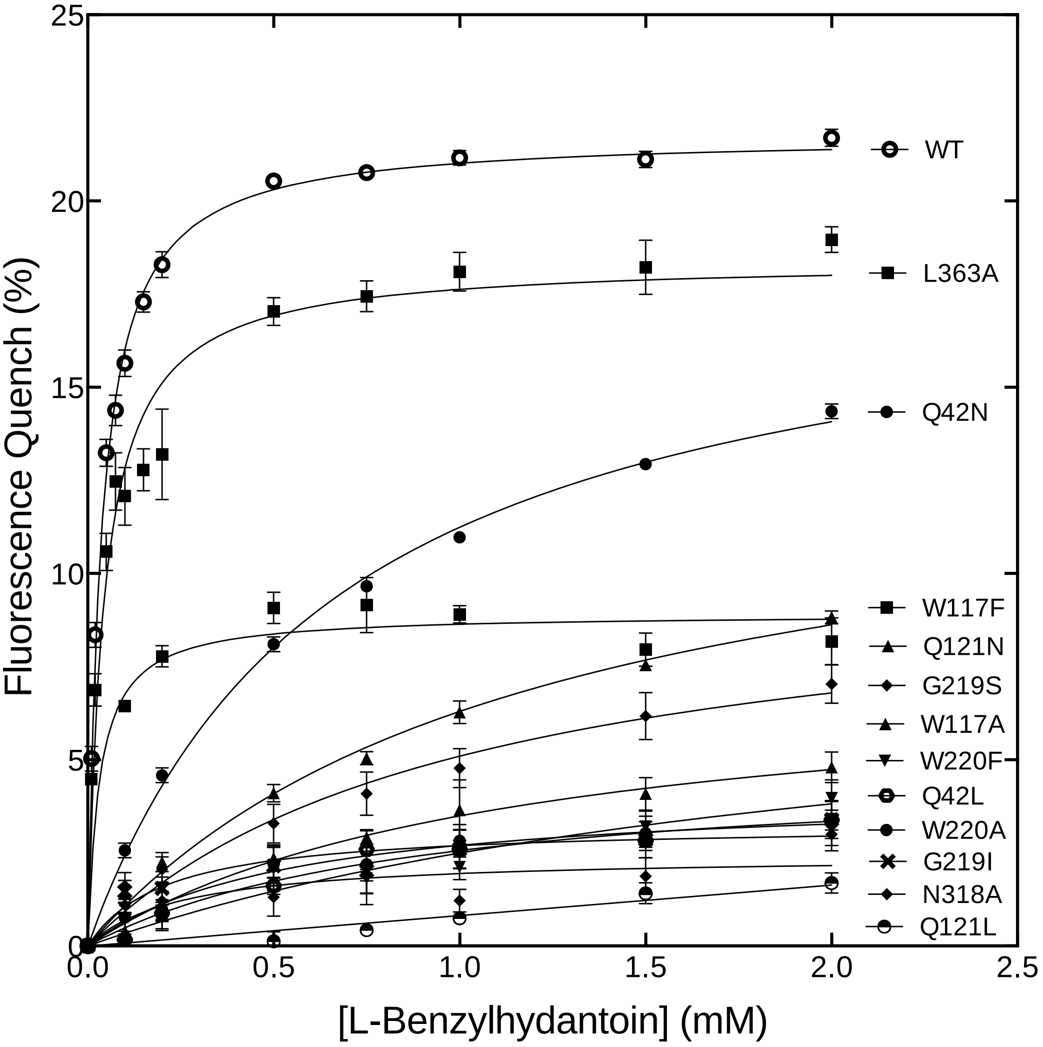

Figure S4. Fluorescence quench curves for binding of L-BH to Mhp1 containing mutations in the ligand binding site. Percentage tryptophan fluorescence quench from steady state measurements seen upon the titration of L-BH up to a concentration of 2 mM with purified wild-type and the indicated mutant Mhp1 proteins (140 µg/ml) in 10 mM Tris pH 7.6, 0.05% DDM, 2% DMSO, 15 mM NaCl and 125 mM choline chloride at 18 °C. Curves were fitted using the Michaelis Menten analysis tool of Graph Pad Prism 6 software. At least triplicate measurements were taken with error bars showing the standard error of the mean. These curves were used to generate the percentage fluorescence quench and binding affinity values given in **Supplementary Table SI**.
