# Supplementary material for: Molecular mechanism of ligand recognition by membrane transport protein, Mhp1
Source: EMBO J. 2014 Jun 21;33(16):1831–44. doi: 10.15252/embj.201387557 (PMC4195764; doi:10.15252/embj.201387557)
Supplement: Supplementary file 5 [file embj0033-1831-sd5.pdf]

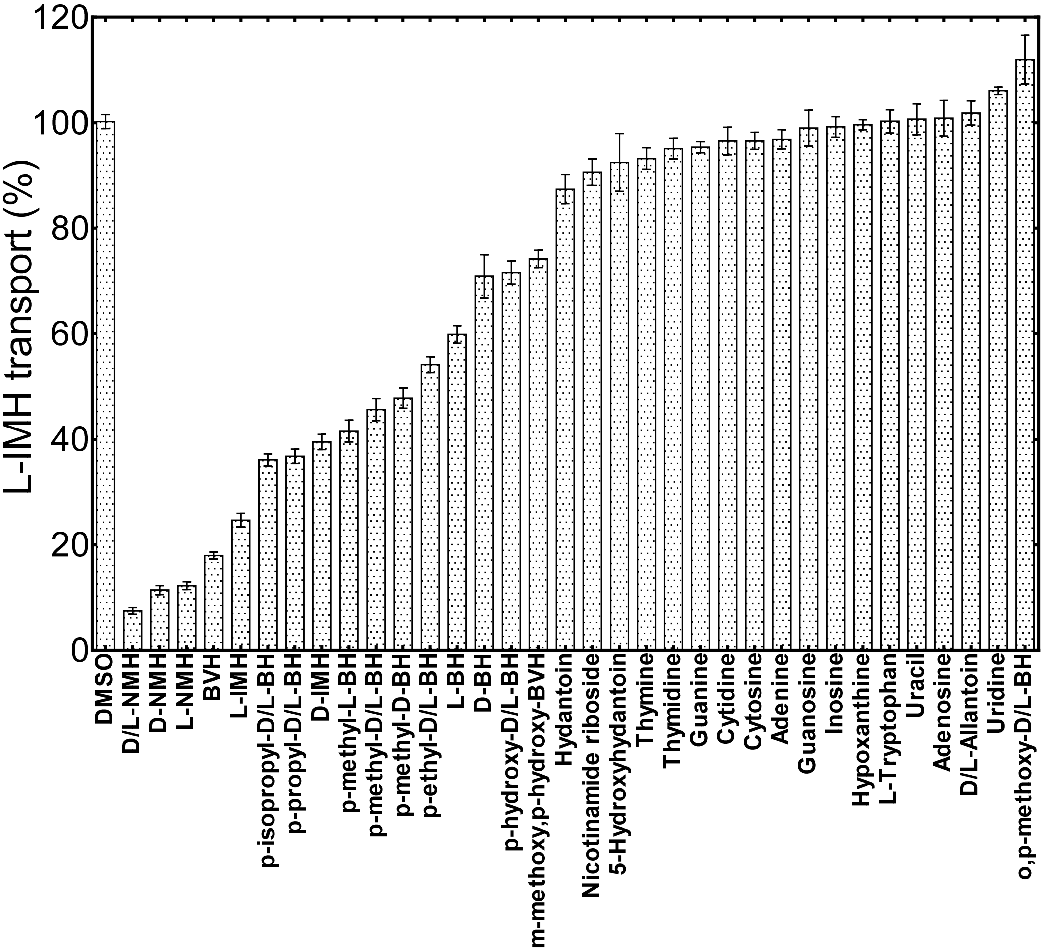

Figure S5. Inhibition of L-IMH uptake into cells by selected compounds. Cells were induced for expression of wild-type Mhp1 (Methods). ^{14}C-L-IMH (50 µM initial concentration) was added to the cells in 150 mM NaCl, 5 mM MES pH 6.6 and the appearance of radioactivity in the cells was measured after 15 sec and 2 min (15 sec data are shown). For competition assays, unlabelled compound (500 µM) was added 3 min beforehand. The mean radioactivity was converted to nmol/mg dry mass at each time point and expressed as percentage of the controls without unlabelled compound. All measurements were repeated at least in triplicate and are shown with error bars denoting standard errors of the mean.
