# Supplementary material for: Molecular mechanism of ligand recognition by membrane transport protein, Mhp1
Source: EMBO J. 2014 Jun 21;33(16):1831–44. doi: 10.15252/embj.201387557 (PMC4195764; doi:10.15252/embj.201387557)
Supplement: Supplementary file 6 [file embj0033-1831-sd6.pdf]

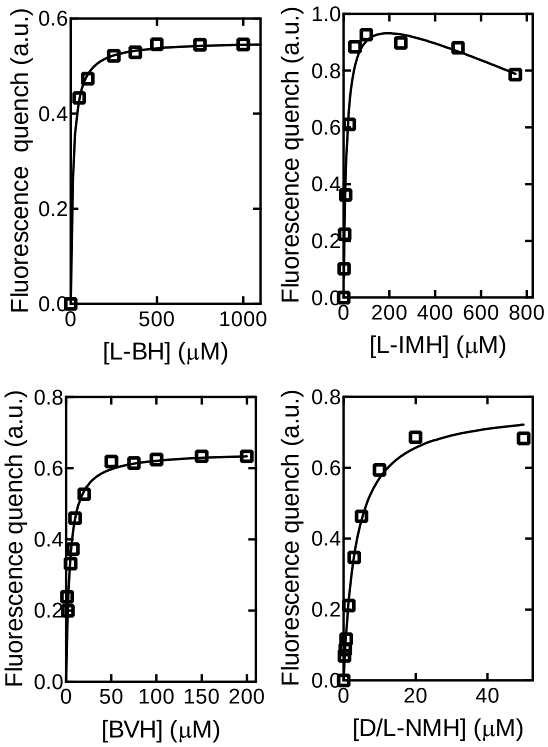

Figure S6. Stopped-flow fluorimetry titration of Mhp1 with ligands. Stopped flow measurements were made of the quench in fluorescence of tryptophan residues in wild-type Mhp1 during titration with the indicated example ligands in the presence of 15 mM NaCl as described in **Supplementary Methods**.
