# Supplementary material for: Molecular mechanism of ligand recognition by membrane transport protein, Mhp1
Source: EMBO J. 2014 Jun 21;33(16):1831–44. doi: 10.15252/embj.201387557 (PMC4195764; doi:10.15252/embj.201387557)
Supplement: Supplementary file 7 [file embj0033-1831-sd7.pdf]

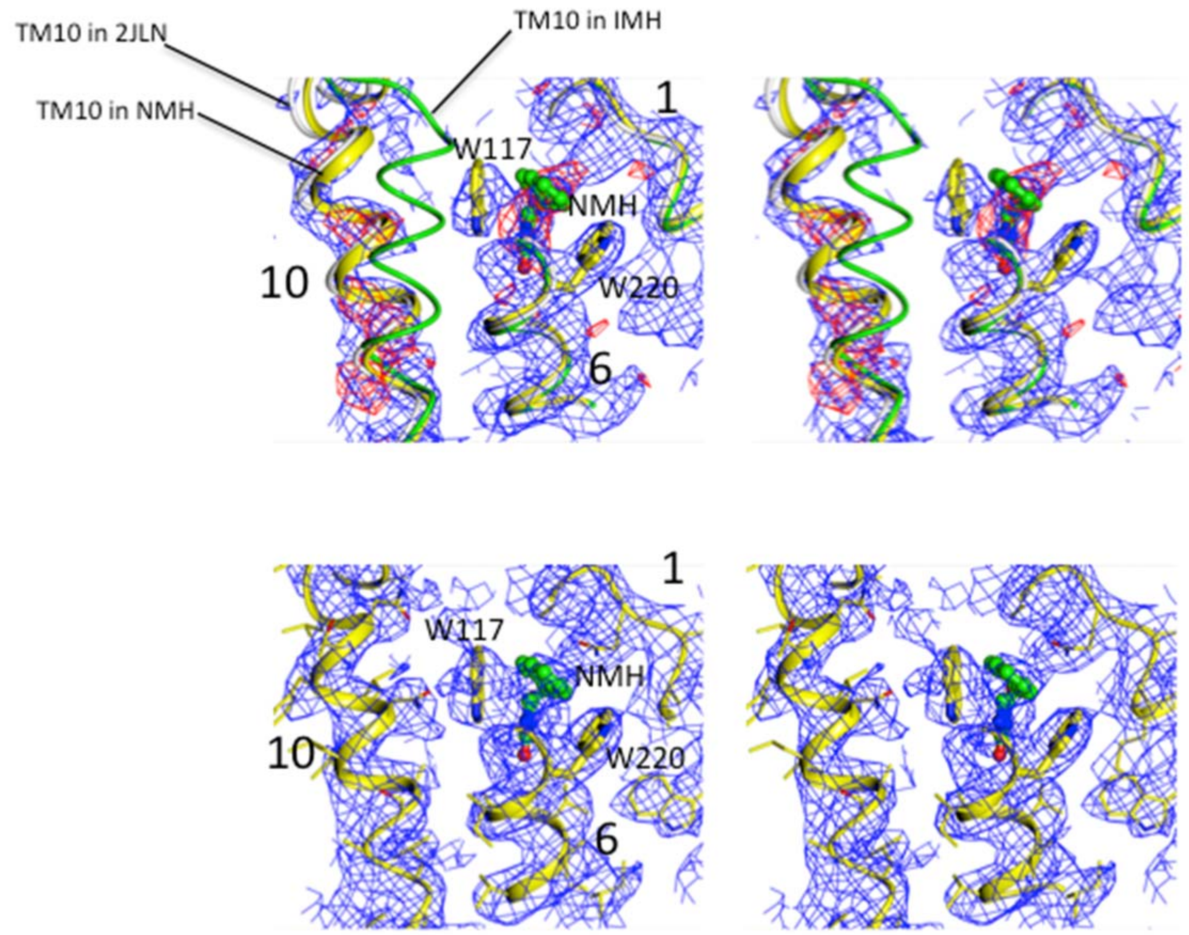

Figure S7. Stereo images showing the position of TMH10 in the L-NMH complex. (Upper) Electron density after initial refinement. The sharpened 2mFo-DFc (blue, 1σ) and mFo-DFc (red, 3σ) maps were calculated based on the IMH complex structure with TMH10 and the ligand omitted from the structure. The final refined models of the NMH (yellow), IMH (green) and the outward-open structure (2JLN; white). These maps show very clearly that the density for TMH10 resembles the outward-open structure rather than the ligand-bound IMH structure. NMH is clearly defined sandwiched between Trp 117 and Trp 220. **(Lower)** A sharpened 2mFo-DFc (blue, 1σ) map based on the final refined structure. While the position of the helix is unambiguous the side-chains are not all well-defined. These have been modelled based on the higher resolution outward-facing structure.
