# Supplementary material for: Molecular mechanism of ligand recognition by membrane transport protein, Mhp1
Source: EMBO J. 2014 Jun 21;33(16):1831–44. doi: 10.15252/embj.201387557 (PMC4195764; doi:10.15252/embj.201387557)
Supplement: Supplementary file 8 [file embj0033-1831-sd8.pdf]

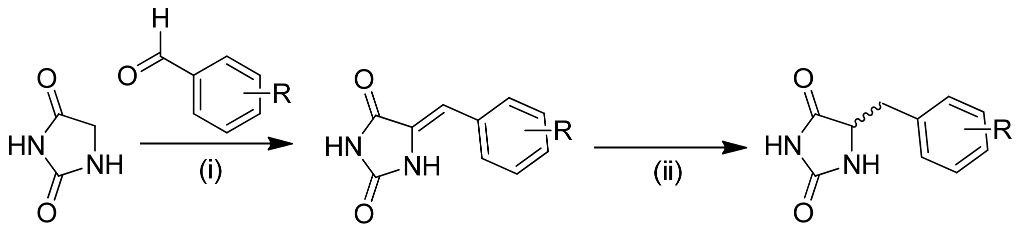

**Figure S8. Synthesis of racemic hydantoin derivatives**. (i) ethanolamine, $NaHCO_3$, EtOH, $H_2O$, 110 °C, 16 h, 35-76%; (ii) $H_2$, Pd/C, EtOH, 16 h, rt, 40-91%.
