# Supplementary material for: Molecular mechanism of ligand recognition by membrane transport protein, Mhp1
Source: EMBO J. 2014 Jun 21;33(16):1831–44. doi: 10.15252/embj.201387557 (PMC4195764; doi:10.15252/embj.201387557)
Supplement: Supplementary file 9 [file embj0033-1831-sd9.pdf]

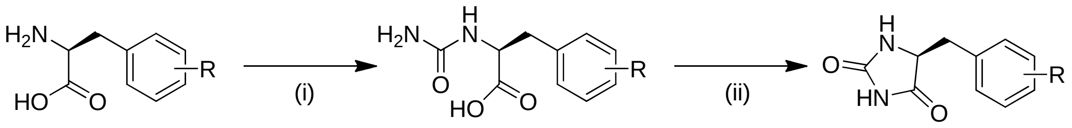


**Figure S9. Synthesis of enantiomerically pure hydantoin derivatives.** (i) KOCN, HCl, $H_2O$, 50-70 °C, 0.5-16 h; (ii) HCl, $H_2O$, reflux, 0.5-16 h.
