# Supplementary material for: Molecular mechanism of ligand recognition by membrane transport protein, Mhp1
Source: EMBO J. 2014 Jun 21;33(16):1831–44. doi: 10.15252/embj.201387557 (PMC4195764; doi:10.15252/embj.201387557)
Supplement: Supplementary file 10 [file embj0033-1831-sd10.pdf]

Table S1. Impairment of hydantoin uptake and binding in mutants of the Mhp1 ligand binding site.

Mutant	^{14}C-L-IMH uptake [a] (%)	Fluorescence quench [b] (1 mM L-BH) (% of WT)	Fluorescence apparent *Kd* [c] BH) (µM)
**WT**	**100** ± 2	**100** ± 1	**40** ± 1
**Q42N**	**83** ± 3	**52** ± 1	**715** ± 63
**Q42L**	**34** ± 2	**14** ± 2	**1011** ± 430
**W117A**	**1** ± 2	**19** ± 4	**1198** ± 383
**W117F**	**63** ± 1	**42** ± 1	**40** ± 10
**Q121N**	**46** ± 2	**31** ± 1	**1232** ± 135
**Q121L**	**28** ± 2	**5** ± 0.3	**ND**
**G219I**	**-1** ± 1	**14** ± 2	**308** ± 131
**G219S**	**51** ± 3	**24** ± 2	**1083** ± 313
**W220A**	**76** ± 3	**15** ± 2	**647** ± 278
**W220F**	**89** ± 3	**12** ± 2	**2329** ± 1249
**N318A**	**39** ± 3	**7** ± 1	**517** ± 339
**L363A**	**85** ± 7	**82** ± 2	**39** ± 4

[a] The accumulation of ^{14}C-L-IMH (50 µM initial concentration) was measured in cells expressing the wild-type or mutant Mhp1 protein in the presence of 150 mM NaCl. [b]For comparative purposes the fluorescence quench data are expressed as a percentage of the wild-type value determined at 1 mM L-BH. [c] Apparent K_d values were calculated by titrating each Mhp1 protein with 0.05-2.0 mM L-BH using the steady state fluorescence quench assay in the presence of 15 mM NaCl (**Methods** and **Fig. S4**), and fitting the data to the Michaelis Menten equation using Graph Pad Prism 6. At least three measurements were taken for each mutant with standard errors of the mean shown. ND denotes 'not determined'.
