# Supplementary material for: Molecular mechanism of ligand recognition by membrane transport protein, Mhp1
Source: EMBO J. 2014 Jun 21;33(16):1831–44. doi: 10.15252/embj.201387557 (PMC4195764; doi:10.15252/embj.201387557)
Supplement: Supplementary file 11 [file embj0033-1831-sd11.pdf]

Supplementary Methods

Small-scale expression of the Mhp1 wild-type and mutated proteins prior to whole cell radiolabeled substrate transport assays. A single colony of *E. coli* BL21(DE3) cells transformed with the pTTQ18-His_6 plasmid containing the gene insert for expressing Mhp1 wild-type or mutated proteins was used to inoculate LB medium (10 ml) supplemented with 100 µg/ml carbenicillin and grown overnight at a temperature of 37 ^oC and with aeration at 200 rpm. An aliquot (4 ml) of the resultant culture was used to inoculate 200 ml of M9 minimal medium (6 g/L Na_2HPO_4, 3 g/L KH_2PO_4, 0.5 g/L NaCl, 5 g/L NH_4Cl) supplemented with 100 µg/ml carbenicillin, 0.4% w/v casamino acids, 20 mM glycerol, 2 mM $MgSO_4.7H_2O$ and 0.2 mM $CaCl_2.2H_2O$ and grown until the A_{600nm} reached 0.3-0.4 (~3.5 h) when the cells were induced with 0.2 mM IPTG. Cells were then grown at 27 ^oC for 12 h post induction and harvested by centrifugation (10 min at 12000 x g, 4 °C).

Whole cell transport assays using ^{14}C-L-indolylmethylhydantoin (L-IMH). Transport assays using intact *E. coli* cells were adapted from the method described by Henderson and MacPherson (Henderson & Macpherson, 1986) and Henderson and Suzuki (Suzuki & Henderson, 2006). Owing to the relatively high background caused by unspecific binding of radioisotope-labelled compound to filters, containers, *etc.* all experiments were compared to the uptake of radioactivity into induced wild-type cells [0.888±0.021 (n=34) nmol/mg per 15 sec s.e.m.] minus the apparent uptake into uninduced cells [0.321±0.016 (n=8) nmol/mg per 15 sec s.e.m.]. This difference was set to 100% for comparative purposes. Harvested cells were washed three times with a buffer containing 150 mM NaCl and 5 mM MES (pH 6.6) and resuspended to a final A_{680nm} of 2 (Henderson & Macpherson, 1986; Suzuki & Henderson, 2006). The uptake of ^{14}C-L-IMH (Patching, 2011) (initially 50 µM) dissolved in water warmed to 25 ^oC was measured at time points of 15 sec and 2 min after its addition to aerated cells in the presence of 20 mM glycerol at 25^oC. The inhibition of 50 µM ^{14}C-L-IMH uptake by putative unlabelled competing compounds was measured by addition of the compounds at a concentration of 500 µM to the cells 3 min prior to the ^{14}C-L-IMH addition. Unlabelled compounds that produced significant inhibition of ^{14}C-L-IMH uptake were then titrated over a concentration range of 1.5-500 µM to test the efficiency of their inhibition and apparent IC_{50} values were subsequently generated using the Graph Pad Prism 6 software. All transport assays were repeated at least in triplicate on at least two separate batches of cells. To correct for any variations in expression of Mhp1 mixed membranes were made by the water-lysis method (Ward et al, 2000; Witholt et al, 1976) and the appearance of Mhp1 checked by Coomassie Blue staining and Western blotting analysis of the proteins separated by SDS-PAGE. Mhp1 protein expression was typically ~20% of the total mixed membrane fraction.

Expression of the Mhp1 wild-type and mutated proteins prior to purification. A single colony of *E. coli* BL21(DE3) cells transformed with the pTTQ18-His_6 plasmid containing the gene insert for expressing Mhp1 wild-type or mutated proteins was used to inoculate LB medium supplemented with 100 μg/μl of carbenicillin and grown at a temperature of 37 ^oC with aeration at 200 rpm until the A_{680nm} reached 1-1.5 (~5-7 h). A 0.1% inoculum was transferred to LB medium supplemented with 100 μg/μl of carbenicillin and grown overnight at 30 ^oC, 200 rpm. For medium scale cultures a 1% inoculum was transferred to 600 ml M9 minimal medium (6 g/L Na_2HPO_4, 3 g/L KH_2PO_4, 0.5 g/L NaCl) supplemented with 100 μg/μl of carbenicillin, 0.2% w/v casamino acids, 20 mM glycerol, 2 mM $MgSO_4.7H_2O$, 0.4 mM $CaCl_2.2H_2O$ and 20 mM NH_4Cl in 2 litre baffled flasks incubated at 200 rpm at 37 ^oC until the A_{680nm} reached 0.3-0.35 (~3.5 h). For large scale preparations cells were grown similarly but in a 30 or 100–liter fermenter. Cells were induced with 0.5 mM IPTG and then grown at 27 ^oC for 5 h post induction before harvesting by centrifugation (16000 x g, 20 min, 4 ^oC). For small scale analyses of expression, mixed membranes were made by the water-lysis method (Ward et al, 2000; Witholt et al, 1976).

For larger scale 6-100 litre cultures (total volume) cell pellets were resuspended in a buffer containing 20 mM Tris, 0.5 mM EDTA, 10% v/v glycerol (pH 7.5) and lysed by explosive decompression using a Constant Systems cell disruptor. The inner and outer membrane fractions were separated using a sucrose gradient of 55-25% (Ward et al, 2000) before resuspension of the inner membrane fraction in Tris buffer (20 mM, pH 7.6), dispensing as aliquots, freezing in liquid nitrogen and storage at -80 ^oC.

Solubilization and purification of His_6-tagged wild-type and mutated Mhp1 protein from the inner membrane prior to biochemical analyses. Preparations of inner membranes with amplified expression of Mhp1 were resuspended to a final concentration of 2-3 mg/ml in solubilization buffer (20 mM Tris pH 8.0, 20 mM imidazole pH 8.0, 300 mM NaCl, 20% v/v glycerol and 1% w/v *n*-dodecyl-β-D-maltoside (DDM)) and mixed for 2 h at 4 ^oC. Insoluble material was removed by ultracentrifugation (100,000 x g, 1 h, 4 ºC) then the soluble supernatant fraction was incubated with Ni-NTA resin for 2 h at 4 ºC with mixing. The Ni-NTA supernatant mixture was transferred to a BioRad column and the unbound fraction was collected. The resin was washed with 100 ml of wash buffer 1 (10 mM Tris pH 8.0, 20 mM imidazole pH 8.0, 10% v/v glycerol and 0.05% DDM) to remove any remaining unbound constituents. The His_6-tagged Mhp1 protein was removed from the column using elution buffer (10 mM Tris pH 8.0, 200 mM imidazole pH 8.0, 2.5% v/v glycerol, 0.05% DDM). The eluted samples were concentrated to 3 ml using a Vivaspin 20 tube molecular weight cut off (MWCO) 100 kDa with centrifugation at 3000 x g, 4 °C. The 3 ml sample was then applied to a BioRad econo-pac 10 DG desalting column to remove the high concentration of imidazole present. When the sample had run into the column 4 ml of wash buffer 2 (10 mM Tris pH 7.6, 2.5% v/v glycerol, 0.05% DDM) was run through the column and the eluting fraction was

collected in a Vivaspin 6 tube MWCO 100 kDa with centrifugation at 4000 x g, 4 °C. The purified eluted protein was concentrated to 5-30 mg/ml, dispensed into aliquots, frozen in liquid nitrogen and stored at -80 ºC.

Protein quantification. Protein concentrations were quantified by the method of Schaffner and Weissman (Schaffner & Weissmann, 1973). SDS-PAGE followed by staining with Coomassie Blue and scanning densitometry were used to measure protein expression and purification accompanied by immunoblotting and chemiluminescence to confirm the presence of the His_6-tagged Mhp1 proteins.

Mhp1 protein expression and purification for co-crystallization with ligands. For crystallization, Mhp1 was cloned into the pWALDO-GFPe plasmid (Drew et al, 2006) to be expressed as a cleavable fusion protein with GFP-His_8. The plasmid was then transformed into BL21 Lemo *E. coli* cells (Wagner et al, 2008). A single colony was used to inoculate 5 ml LB medium containing antibiotics (50 ug/ml kanamycin, 34 µg/ml chloramphenicol). After incubating for 30 h at 37 °C with shaking at 225 rpm, 150 mL LB medium containing antibiotics were inoculated with 1.5 mL of the 5-mL culture and then incubated for 12-16 h at 37 °C and 225 rpm. 10 ml of this culture was used to inoculate 1 L of the autoinduction medium PASM-5052 (Studier, 2005) and the cells grown at 37 °C and 225 rpm until the A_{600nm} reached 0.1-0.2, upon which the temperature was lowered to 25 °C. When the culture reached an A_{600nm} of 0.5, 0.4 mM IPTG was added to induce expression. The culture was harvested 22-24 h after IPTG addition by centrifugation at 5000 x g for 10 min. All subsequent steps were performed on ice or at 4 °C.

The cell pellet was resuspended in PBS pH 7.4, 1 mM Pefabloc, 1 mM $MgCl_2$, 125 mg/L DNase I (from bovine pancreas) and cells were lyzed by explosive decompression using a Constant Systems cell disruptor followed by centrifugation at 24,000 x g for 12 min. The supernatant was recovered and membranes were sedimented by centrifugation at 130,000 x g for 1 h. The pelleted membranes were then resuspended in 30 mL PBS pH 7.4, 0.5 mM Pefabloc, frozen in liquid nitrogen and stored at -80 °C.

Frozen membranes were thawed and diluted with phosphate-buffered saline (PBS) pH 7.4, 0.2 mM Pefabloc to 3-4 mg/mL total protein concentration (determined with the BCA assay). The membranes were then solubilized by adding 1% (w/v) nonyl maltoside (Anatrace, NM) with stirring for 1 h, followed by centrifugation at 130,000 x g for 1 h to remove insoluble material. The amount of GFP fusion protein in the soluble fraction was determined by measuring the GFP fluorescence intensity as described (Drew et al, 2006). The C-terminally tagged fusion protein was bound to Ni-NTA Superflow IMAC resin (Qiagen) in the presence of 20 mM imidazole while slowly stirring in a beaker for 2-3 h. 0.5-1 mL of resin, equilibrated in PBS pH 7.4, 0.5% NM, 0.2 mM Pefabloc, 20 mM imidazole, was added to the solubilized membranes per mg of GFP. The IMAC resin was then

transferred into a gravity flow column, washed with 2 column volumes (CV) of PBS, pH 7.4, 0.5 % (w/v) NM, 0.2 mM Pefabloc, 20 mM imidazole, then 2 CV PBS pH 7.4, 0.5 % (w/v) NM, 30 mM imidazole and eluted with PBS pH 7.4, 0.5% (w/v) NM, 250 mM imidazole.

The fused GFP was cleaved by incubating with an equimolar amount of His-tagged TEV protease in the presence of 5 mM β-mercaptoethanol and 5 mM sodium citrate for 12-16 h while dialyzing against buffer (20 mM Tris pH 8.0, 150 mM NaCl, 0.5% (w/v) NM, 2.5% glycerol). The His-tagged cleaved GFP and the His-tagged TEV protease were subsequently removed by reverse IMAC. The imidazole concentration in the dialyzed sample was adjusted to 15 mM and then the sample was passed through a 5-ml HisTrap HP column (GE Healthcare) equilibrated with dialysis buffer with 15 mM imidazole. The column was then washed with 2 CV of the same buffer. The column flow-through and wash fractions containing pure Mhp1 were combined, the buffer was exchanged to 10 mM Tris pH 8.0, 0.5% (w/v) NM, 2.5% (v/v) glycerol, and the protein was concentrated with a centrifugal ultrafiltration device (Vivaspin 20, 100,000 MWCO) to 20-30 mg/ml.

X-ray diffraction analyses. Mhp1 was crystallized by the hanging-drop vapor diffusion method at 20 °C as described previously (Shimamura et al, 2008; Weyand et al, 2008). 1 μl of protein solution was mixed with 1-1.5 μl of reservoir solution containing 0.1 M sodium phosphate pH 7.0, 0.1 M NaCl and 27-32% (v/v) PEG 300. For co-crystallization of Mhp1 with IMH, BH and NMH saturated solutions of the ligands in reservoir solution were prepared and then 1-1.5 μl was directly added to the protein drop. For co-crystallization of Mhp1 with BVH, a stock solution of the ligand in PEG 300 was prepared and then 5-10 mM was added to the concentrated protein solution and incubated at 20 °C for 30 min before crystallization.

Crystallographic Data Collection and Structure Solution

Crystal structure of Mhp1 complexed with L-IMH.

A crystal was mounted on a mesh loop and dehydrated using the humidity controller HC1 device (Sanchez-Weatherby et al, 2009) by reducing the humidity from 96% to 80% at a rate of 1% per minute. After leaving for 10 minutes at 80% the crystal was then flash frozen in liquid nitrogen and data were collected at beamline I02 of Diamond Light Source. The data were processed to a resolution of 3.4 Å using XDS (Kabsch, 2010) interfaced through Xia2 (Winter, 2010) with further processing using the CCP4 suite (Collaborative Computational Project Number 4, 1994). The space group and cell dimensions were similar to those observed previously for the outward-facing conformations of the protein (Weyand et al, 2008). The structure was solved by molecular replacement in Phaser (McCoy et al, 2007) using the outward-facing open conformation of the protein (2JLN (Weyand et al, 2008)) as the search model. The structure was refined using the Phenix package (Adams et al, 2010) with restraints on the dihedral angles derived from the higher resolution structure. One B-factor was

assigned per residue and the whole protein was considered as one TLS group (Winn et al, 2001). Manual rebuilding was carried out in O (Jones et al, 1997) or Coot (Emsley & Cowtan, 2004) with reference to sharpened maps. Following adjustments of some side-chains and the main chain around TM10 the L-IMH ligand was inserted into clear density in the center of the protein. A sodium ion was inserted in the same position as the ion in 2JLN and refined with restraints.

Crystal structure of Mhp1 complexed with NMH.

Data were collected from a crystal that was dehydrated as described above and flash-frozen in liquid nitrogen. The data were processed and scaled using the HKL2000 suite (Otwinowski et al, 1997) with merging and further processing carried out using programs from the CCP4 suite (Collaborative Computational Project Number 4, 1994). The structure was initially refined as described above starting from the refined structure of the IMH complex, but with the ligand omitted, positioned in the unit cell as seen for the benzyl-hydantoin structure (2JLO). Upon inspection of the electron density maps it was evident that TMH10 did not adopt the position taken in the IMH structure but rather the position seen in the unbound 2JLN. Test refinements were therefore carried out starting with both the IMH structure and 2JLN correctly position in the unit cell, with the N-terminal part of TMH10 omitted from both structures. These showed the R-free to be 2% lower for the refinement based on the IMH structure than for that based on 2JLN. Further refinement was carried out from the IMH structure replacing the N-terminal part of TMH10 with the equivalent residues from 2JLN (residues 347-374). The ligand was clearly defined in the binding site. As a) an enantiomeric mixture of the L and D forms of NMH was added to the crystallization mixture; b) no discrimination could be observed in the inhibition assay; c) the electron density was consistent with both enantiomers and d) no difference was observed in the R_{free} between refinements carried out with the individual compounds the structure was refined with both enantiomers present, each with an occupancy of 0.5.

Crystal structure of Mhp1 complexed with L-BH and BVH

Data for the L-BH and BVH were also collected and processed as described for the NMH complex though no dehydration of the crystals was first carried out. Again the correctly position IMH structure was used as the starting model for refinement. Anomalous difference maps for the BVH structure showed the position of the bromine atom. During refinement against the data from BVH it became apparent that there was residual electron density in the same position as for TMH10 in the NMH structure. As this could only be observed at low contour levels and no improvement was made to the R_{free} when modeled as multiple conformations, only the predominant conformation was modeled. Data were also collected from a complex with the equivalent iodo derivative (IVH) on the iodine edge at a resolution of 3.7 Å. Since the anomalous difference maps clearly showed the iodine to bind in the same position as the bromine of BVH this structure was not refined. It was noted, however, from the

electron density maps that the TMH10 helix was even more often in the open position than seen for BVH.

Structural Analysis Superpositions were carried out in Lsqman (Kleywegt & Jones, 1994) on the respective bundle helices. The superpositions were performed so that only C_a pairs which were less than 3.8 Å apart were included in the calculation. Figures showing the structure were drawn using Pymol (Delano, 2002) except those showing electron density, which were made using the CCP4mg (Potterton et al, 2004).

Steady-state tryptophan fluorescence to measure binding of compounds to purified wild-type Mhp1. Steady-state fluorescence spectroscopy was performed using a PTI spectrofluorometer. Measurements used 140 μg/ml of purified Mhp1(His_6) protein in 10 mM Tris pH 7.6, 0.05% DDM, 2% DMSO, 125 mM choline chloride, 15 mM NaCl at a temperature of 18 ^oC. Samples were excited at 295 nm and fluorescence emission was measured at 330-335 nm. Microliter additions of each compound dissolved in 100% DMSO were added over a concentration range of 0-2 mM and accounted for <2% of the final volume. Samples were mixed for 2 min after each compound addition before the fluorescence emission was measured. The data were analyzed to determine apparent K_d and F_{max} values using the Michaelis Menten analysis tool of Graph Pad Prism 6 software.

Stopped-flow fluorescence measurements of ligand binding to Mhp1. Stopped-flow fluorescence measurements were performed using a SX.18MV-R stopped-flow reaction analyzer from Applied PhotoPhysics. The assembly reaction was initiated by rapid mixing of a buffer solution containing Mhp1 in 125 mM choline chloride, 15 mM NaCl, 10 mM Tris-HCl, pH 7.6, 0.05% DDM, 2% DMSO with the same buffer containing instead an hydantoin in a ratio of 1:1 (v/v). The mixing dead time was less than 5 ms and the temperature was 18 ^oC. The time course of complex formation was measured by following Trp fluorescence intensity change using an excitation wavelength of 280 nm and a cut off filter of 330 nm. Each reaction curve represented an average of at least eight experimental runs depending on the sensitivity of the fluorescence signals. The overall rate constant for the fast or slow process of the complex formation was analyzed using a single exponential function $F(t)=F_0+A*(exp(-k_{obs}*t))$. Data analyses were performed using the software Gnuplot, Sigmaplot 7.0 and GraphPad Prism 6. Error bars represent the results of at least three experiments, and curves were fitted to all data points using GraphPad Prism 6 software.

Molecular dynamics simulations of free ligands in aqueous solution. The ligands L-BH, L-IMH, and L-NMH were parameterized with the OPLS-AA force field (Rizzo & Jorgensen, 1999) using atom types of chemically equivalent groups and partial charges as to match the experimental water-octanol partition coefficients when known (Iorga & Beckstein, unpublished data). Parameters of L-

BH, L-IMH, and L-NMH were deposited in the *Ligandbook* small molecule force field parameter repository (http://ligandbook.icsn.cnrs-gif.fr/). Equilibrium MD simulations of each ligand molecule in water (TIP4P water model) (Jorgensen & Madura, 1985) were performed at T=300 K and P=1 bar with Gromacs 4.5.3 (Hess et al, 2008) for 100 ns (L-BH, L-NMH) or 200 ns (L-IMH). Simulations were performed under periodic boundary conditions in rhombic dodecahedral simulation boxes, with a minimum solute-box face distance of 1 nm. The temperature was controlled with the Langevin dynamics integrator (integration time step 2 fs, friction coefficient set to particle mass/0.1 ps) and simulations were conducted at constant pressure with Berendsen's weak coupling scheme (Berendsen et al, 1984) with relaxation time constant 1 ps and the compressibility of water, 4.6×10^{-5} bar^{-1}. Electrostatic interactions were computed with the SPME method (Essman et al, 1995) on a 0.12 nm FFT grid and 4th order spline interpolation and a real space cut-off of 1 nm. Lennard-Jones interactions were calculated up to a cut-off of 1 nm while energy and pressure were corrected for dispersion interactions beyond the cut-off. Bonds containing hydrogen atoms were constrained using the P-LINCS algorithm (Hess, 2008) (fourth order expansion with a single iteration).

The dihedral angles χ_1 (between the hydantoin C-5 and the bridging C atom) and χ_2 (between the bridging C atom and the first C on the aromatic ring connected to the bridging C) determine the overall conformation of the molecule. (The conformation of L-BH is determined by χ_1 alone due to the symmetry of the benzene ring.) $\chi_1$and χ_2 were analyzed at intervals of 1 ps with the MDAnalysis tool kit (Michaud-Agrawal et al, 2011). The resulting time series was histogrammed as $p(\chi_1, \chi_2)$ using 1º-bins and the conformational free energy landscape calculated as $W(\chi_1, \chi_2) = -kT \ln p(\chi_1, \chi_2)$. Stable or metastable conformations are detected as local minima of $W(\chi_1, \chi_2)$ (or $W(\chi_1)$ for L-BH).

The probability to observe a given conformation was calculated as the probability to observe the local minimum, i.e. the range of dihedral angles between the adjacent maxima. For instance, the "folded" or "U-shaped" conformation of L-BH was calculated as $p_U = p(0° < \chi_1 < 120°) = \int_{0°}^{120°} \exp[-W(\chi_1)/kT]\, d\chi_1 / \int_{-180°}^{180°} \exp[-W(\chi_1)/kT]\, d\chi_1$. Free energy differences between conformations were calculated from logarithms of ratios between population probabilities; e.g. L-BH's folded conformation *vs* all other (extended) ones: $G_U - G_{other} = -kT \ln p_U/(1-p_U)$.

The parameterization for L-BH was validated by comparison to experimental conformer distributions derived from NMR (Kleinpeter, 1997). In experiments, L-BH shows an unexpected preference for the folded over conformation (g+) that has been attributed to non-bonded intramolecular interactions between the benzene substituent and the dipole of hydantoin ring. The simulations of L-BH in water reproduce the experimentally observed preference for the folded conformation (+60º, g+) and also show that the anti (–180º) conformer is the least stable one (**Supplementary Fig. S2**). The ±60º population is stabilized over the –180º population by –2.24 kT, which is in good agreement with the value of –1.66 kT calculated from the experimental population data.

Molecular dynamics simulations of ligands bound to Mhp1. Simulations of the occluded conformation of Mhp1 with L-BH and L-IMH ligands were performed as described previously (Shimamura et al, 2010) with the following differences. As a starting structure the IMH-bound structure was used with L-IMH in its extended conformation. Simulations with L-BH started with either the folded over (U-shaped) conformation (dihedral angle χ_1=+60°, g^+) as seen in the small molecule crystal structure and was modeled in the 2JLO structure (Weyand et al, 2008), or in an extended conformation (χ_1=–60°, g^-). The L-BH ligands were modeled into the binding site either by superimposing 2JLN+BH onto the IMH-bound structure (g^+) or by superimposing the hydantoin ring of L-BH onto the one of IMH (g^-). The OPLS-AA force field (Jensen & Jorgensen, 2006; Kaminski et al, 2001; Rizzo & Jorgensen, 1999) was used for the protein atoms and ions together with the TIP4P water model (Jorgensen & Madura, 1985). Mhp1 was simulated in a 1-palmitoyl-2-oleoylphosphatidylcholine (POPC) model membrane, using the Ulmschneider OPLS-UA parameters (Ulmschneider & Ulmschneider, 2009) available from *Lipidbook* (Domański et al, 2010) (http:///lipidbook.bioch.ox.ac.uk). The parameters for L-IMH and L-BH derived for the free solvent simulations were also used for the ligand binding simulations. In all simulations a sodium ion occupied the Na2 site. A simulation system consisted of approximately 83,000 atoms in an orthorhombic simulation cell of ~92 Å × 92 Å × 96 Å, comprising Mhp1 (residues R10–G470), the ligand, ~220 POPC and ~16,000 water molecules together with 28 sodium and 35 chloride ions for a free ion concentration of ~100 mM.

Simulations were performed with Gromacs 4.5.3 (Hess, 2008) at a constant temperature of 310 K and pressure 1 bar using the velocity rescaling algorithm for the thermostat (time constant 0.1 ps) (41) and semi-isotropic weak coupling for the barostat (time constant 1.0 ps, compressibility 4.6×10^{-5} bar^{-1}) (Bussi et al, 2007). Long range corrections for energy and pressure were applied (Hess et al, 2008). Lennard-Jones interactions were cut off at 10 Å while electrostatic interactions were handled by the SPME method (Essman et al, 1995) that computes Coulomb interactions in real space up to a cutoff of 10 Å and long range interactions beyond the cutoff in reciprocal space with fast Fourier transforms on a grid with spacing 1.2 Å and fourth order splines for fitting of the charge density. Bonds to hydrogen atoms were constrained with the P-LINCS algorithm (Hess, 2008) or SETTLE (for water molecules) (Miyamoto & Kollman, 1992). The grid-based neighbour list was updated every 5 steps. The classical equations of motions were integrated with a leap frog integrator and a time step of 2 fs. Conformations were saved every 1 ps for analysis.

Three sets of simulations were carried out: (1) L-BH starting from an extended conformation [5FH(g^-)MD_001 – 5FH(g^-)MD_003]; (2) L-BH starting from the folded over conformation [5FH(g^+)MD_001 – 5FH(g^+)MD_004]; and (3) L-IMH starting from the extended conformation

[IMH(g^{-})MD_001 – IMH(g^{-})MD_001]. At least three repeat simulations were carried out for each set and the simulated time ranged from 14 ns to 500 ns (**Supplementary Fig. S3**).

Hydrogen bonds were detected by a geometric criterion, using the Gromacs tool g_hbond. A hydrogen bond D–H···A was recorded for any time step in the simulation where the distance between the donor and acceptor heavy atoms was ≤ 3.5 Å and the angle between A,D, and H was < 30° (a linear hydrogen bond would have 0°). OH and NH were taken as donors and only O as acceptors. For all hydrogen bonds their occupancy was calculated, i.e. the fraction of simulation time that the bond existed. If multiple water molecules were seen to bind to a particular ligand heavy atom then all these ligand-water bonds were added as water molecules are chemically indistinguishable. As sometimes multiple water molecules were bonded at the same time, some occupancies can exceed 1. Only hydrogen bonds with occupancies > 0.2 are reported here.

Chemical syntheses

The principles of methods for syntheses of racemic mixtures or enantiometrically pure hydantoin derivatives are illustrated in **Supplementary Figs. S8 and S9**.

General information and instrumentation. All reagents obtained from commercial suppliers (e.g. Aldrich) were used without further purification. All solvents were distilled before use or obtained dry from commercial suppliers; petrol refers to petroleum ether (bp. 40-60 °C). Analytical TLC was performed using silica gel pre-coated plates (Merck) and visualized using UV irradiation. Flash column chromatography was carried out on silica gel 60 (230-400 mesh, Merck). Solvents were removed under reduced pressure using a Büchi rotary evaporator at diaphragm pump pressure. Samples were freed of remaining traces of solvents under high vacuum.

^{1}H and ^{13}C NMR spectra were measured on a Bruker DPX300 Fourier transform spectrometer or a Bruker Avance 500 using an internal deuterium lock. Chemical shifts are reported in parts per million (ppm) downfield from TMS in δ units and coupling constants (*J*) are given in hertz (Hz). TMS is defined as 0 ppm for ^{1}H NMR spectra and the center line of the triplet of $CDCl_3$ is defined as 77.10 ppm for ^{13}C NMR spectra. When displaying the ^{1}H NMR data the following abbreviations are used; s = singlet, d = doublet, dd = doublet of doublets, t = triplet, q = quartet, m = multiplet. ^{1}H and ^{13}C signal assignment is based on HMQC and HMBC spectra analysis where appropriate.

Infrared (IR) spectra were recorded as thin films using sodium chloride plates or solid samples on a Perkin Elmer Spectrum One FT-IR spectrophotometer. Vibrational frequencies are reported in wavenumbers (cm^{-1}).

Mass spectra (HRMS) were recorded in house on a Micromass GCT Premier instrument, using electron impact ionization (EI) or a Bruker Daltonics micrOTOF, using electron spray ionization (ESI). All quoted masses refer to the ^{79}Br isotope. Elemental analysis was performed in house using a Carlo Erba 1108 Elemental Analyzer. Determinations of halogens and sulfur were carried out using the Schoniger Oxygen Flask combustion method followed by the relevant titration for the particular halogen.

HPLC analyses (Method A) were performed using a Dionex HPLC system with a Phenomenex Hyperclone C18, 250 x 4.6 mm, 5 micron column and diode array as a detector. A gradient of water and acetonitrile (5-95 %) was used as solvent at a flow rate of 1 ml/min over 30 min.

HPLC analyses (Method B) were carried out on an Agilent 1290 Infinity system using a Supelco Ascentis Express C18, 50 x 2.1 mm, 2.7 micron column and diode array as a detector. A gradient of water and acetonitrile (5-95%) was used at a flow rate of 0.5 ml/min over 3 min.

Melting points were determined on a Reichert Hot Stage, a Griffin Melting Point apparatus or a STUART Melting Point Apparatus. Melting points obtained were uncorrected.

General Method A (based on the procedure of Thenmozhiyal et al. (Thenmozhiyal et al, 2004))*: Synthesis of compounds* ***BVH*** *and* ***m-methoxy,p-hydroxy-BVH***

Hydantoin (500 mg, 5.00 mmol) was dissolved in water (10 ml) at 70 ˚C with stirring. After complete dissolution, the pH of the mixture was adjusted to 7.0 by the addition of saturated $NaHCO_3$ solution. Ethanolamine (0.45 ml) was added to the reaction mixture and the temperature raised to 120 ˚C after addition of the aldehyde (5.00 mmol) dissolved in EtOH (10 ml). After refluxing for 16 h a precipitate was isolated. This was washed with 1:5 EtOH/water (20 ml) to give the title compound.

General Method B (based on the procedure of Takuma and Watanabe (Takuma & Watanabe, 2001) *and used in combination with the preceding General Method A): Synthesis of compounds* ***D/L-NMH, D/L-IMH, p-isopropyl-D/L-BH, p-propyl-D/L-BH, p-methyl-D/L-BH, p-ethyl-D/L-BH and p-propyl-D/L-BH***

To a solution of the methylene hydantoin (1.0 equivalent) in MeOH (20 ml) was added NaOAc (3 equivalents) and 10% Pd/C (10 equivalents). The mixture was stirred at room temperature under an atmosphere of hydrogen for 23 h. The resulting black solution was filtered through celite. Removal of solvent *in vacuo* followed by recrystallization from EtOH gave the title compound.

Characterization of compounds

5-(Naphthalen-2-ylmethyl)imidazolidine-2,4-dione (Takuma & Watanabe, 2001) **(D/L-NMH):** This compound was synthesized from hydantoin and the relevant aldehyde according to the procedures of general methods A and B (59%). **mp** >250 °C. **TLC** R_f 0.10 (Petrol/EtOAc; 1:1). **HPLC** (method A) T_r = 29.94 (91% rel. area). **^{1}H NMR** (300 MHz, DMSO-d_6) δ = 7.95 (1H, s), 7.80-7.89 (2H, m), 7.70 (1H, s), 7.47-7.52 (2H, m), 7.37 (1H, dd, *J* = 1.1, 7.1), 4.42 (1H, t, *J* = 5.0), 3.41 (1H, br s, NH), 3.12 (2H, dd, *J* = 5.0, 9.9). **^{13}C NMR** (75 MHz, DMSO-d_6) δ = 166.0, 156.2, 133.8, 133.2, 132.3, 128.5, 128.4, 126.0, 58.8, 37.1. **IR** ν_{max}/cm^{-1} (neat) 3252, 2937, 2858, 2748, 2394, 1927, 1738, 1600, 1508. **ESI-MS** *m/z* 241.0 (100% MH^+); (Found MH^+, 241.0974 $C_{14}H_{12}N_2O_2$ requires *MH* 241.0977).

(R)-5-(Naphthalen-2-ylmethyl)imidazolidine-2,4-dione (Kawaguchi et al, 2000) **(D-NMH):** This compound was synthesized based on a method by Patching (Patching, 2011). A mixture of 3-(2-naphthyl)-*D*-alanine (100 mg, 0.46 mmol) and potassium cyanate (75.4 mg, 0.93 mmol) in water (30 ml) was stirred and heated at 65-70 °C overnight. The resultant solution was concentrated under vacuum to a volume of ~ 5 ml then 1 M HCl was added dropwise with stirring at room temperature until the precipitate remained in solution. The solid was collected by filtration, washed with water

then dried under vacuum over P_2O_5 to give 3-(2-naphthyl)-*D*-alanylhydantoic acid (78 mg, 0.30 mmol, 66%) as a white powder. **mp** 216-218 °C. **^{1}H NMR** (500 MHz, DMSO-d_6) δ = 7.87-7.34 (7H, m, 7 x Ar-H), 6.18 (1H, d, *J* = 8.5 Hz, NH), 5.59 (2H, s, NH_2), 4.42 (1H, m, *J* = 5 Hz and 8 Hz, CH), 3.08 (2H, m, *J* = 5 Hz, 8 Hz, 14 Hz and 68 Hz, CH_2); **^{13}C NMR** (125 MHz, DMSO-d_6) δ = 174.0 (CO_2H), 158.1 ($CONH_2$), 135.2, 132.9, 131.8, (127.7, 127.6, 127.4 – 5xC), 126.0, 125.5, 53.7 (CH), 37.8 (CH_2). **ESI-MS** (ES^+/TOF) *m/z* 281.1 (MNa^+), 259.1 (MH^+); HRMS (ES^+/TOF) *m/z* calcd for $C_{14}H_{14}N_2O_3$ + Na^+ = 281.0897, found 281.0885 and for $C_{14}H_{14}N_2O_3$ + H^+ = 259.1077, found 259.1078. 70 mg (0.27 mmol) of the solid in 2 M HCl (50 ml) was stirred and heated under reflux overnight. The solution was allowed to cool to room temperature resulting in formation of a fluffy pale precipitate which was collected by filtration, washed with water then dried under vacuum over P_2O_5 to give **D-NMH** (56 mg, 0.23 mmol, 85%) as pale crystals. **mp** 208-210 °C. **^{1}H NMR** (500 MHz, DMSO-d_6) δ = 10.41 (1H, s, NH-3), 7.96 (1H, s, NH-1), 7.87-7.35 (7H, m, Ar-H), 4.41 (1H, m, *J* = 5.5 Hz and 1 Hz, CH), 3.09 (2H, m, *J* = 5 Hz, 5.5 Hz, 14 Hz and 31 Hz, CH_2). **^{13}C NMR** (125 MHz, DMSO-d_6) δ = 175.2 (C-2), 157.1 (C-4), 133.5, 132.8, 131.9, (128.1, 127.5 – 5 x C), 126.0, 125.6, 58.5 (C-5), 36.7 (C-6). MS (ES^+/TOF) **ESI-MS** *m/z* 263.1 (MNa^+), 241.1 (MH^+); HRMS (ES^+/TOF) *m/z* calcd for $C_{14}H_{12}N_2O_2$ + Na^+ = 263.0791, found 263.0786 and for $C_{14}H_{12}N_2O_2$ + H^+ = 241.0972, found 241.0960.

(S)-5-(Naphthalen-2-ylmethyl)imidazolidine-2,4-dione (L-NMH): This compound was synthesized based on a method by Patching (Patching, 2011). A mixture of 3-(2-naphthyl)-*L*-alanine (100 mg, 0.46 mmol) and potassium cyanate (75.4 mg, 0.93 mmol) in water (30 ml) was stirred and heated at 65-70 °C overnight. The resultant solution was concentrated under vacuum to a volume of ~ 5 ml then 1 M HCl was added dropwise with stirring at room temperature until the precipitate remained in solution. The solid was collected by filtration, washed with water then dried under vacuum over P_2O_5 to give 3-(2-naphthyl)-*L*-alanylhydantoic acid (72 mg, 0.28 mmol, 61%) as a white powder. **mp** 222-224 °C. **^{1}H NMR** (500 MHz, DMSO-d_6) δ =7.87-7.35 (7H, m, 7 x Ar-H), 6.18 (1H, d, *J* = 8.5 Hz, NH), 5.59 (2H, s, NH_2), 4.43 (1H, m, *J* = 5 Hz and 8 Hz, CH), 3.09 (2H, m, *J* = 5 Hz, 8 Hz, 14 Hz and 68 Hz, CH_2). **^{13}C NMR** (125 MHz, DMSO-d_6) δ = 173.9 (CO_2H), 158.1 ($CONH_2$), 135.2, 132.9, 131.9, (127.7, 127.6, 127.5 – 5xC), 126.0, 125.5, 53.7 (CH), 37.8 (CH_2). **ESI-MS** (ES^+/TOF) *m/z* 281.1 (MNa^+), 259.1 (MH^+); HRMS (ES^+/TOF) *m/z* calcd for $C_{14}H_{14}N_2O_3$ + Na^+ = 281.0897, found 281.0888; MS (ES^-/TOF) 257.1 (M-H). 72 mg (0.28 mmol) of the solid in 2 M HCl (50 ml) was stirred and heated under reflux overnight. The solution was allowed to cool to room temperature resulting in formation of a fluffy pale precipitate which was collected by filtration, washed with water then dried under vacuum over P_2O_5 to give **L-NMH** (48.5 mg, 0.20 mmol, 71%) as pale crystals. **mp** 226-228 °C. **^{1}H NMR** (500 MHz, DMSO-d_6) δ =10.41 (1H, s, NH-3), 7.96 (1H, s, NH-1), 7.87-7.35 (7H, m, Ar-H), 4.41 (1H, m, *J* = 5.5 Hz and 1 Hz, CH), 3.09 (2H, m, *J* = 5 Hz, 5.5 Hz, 14 Hz and 31

Hz, CH_2). **^{13}C NMR** (125 MHz, DMSO-d_6) δ = 175.2 (C-2), 157.1 (C-4), 133.5, 132.8, 131.9, (128.1, 127.5 – 5 x C), 126.0, 125.6, 58.5 (C-5), 36.7 (C-6). **ESI-MS** (ES^+/TOF) *m/z* 263.1 (MNa^+), 241.1 (MH^+); HRMS (ES^+/TOF) *m/z* calcd for $C_{14}H_{12}N_2O_2$ + Na^+ = 263.0791, found 263.0796 and for $C_{14}H_{12}N_2O_2$ + H^+ = 241.0972, found 241.0968.

[2-^{14}C]-(S)-5-(Naphthalen-2-ylmethyl)imidazolidine-2,4-dione ([2-^{14}C]-L-NMH): A ^{14}C-labelled version of compound **L-NMH** was prepared by using the same procedure described above for the unlabeled compound except that 500 μCi [^{14}C]potassium cyanate (specific activity 40 mCi/mmol; American Radiolabelled Chemicals) in water (1 ml) was included along with the unlabelled potassium cyanate in the first reaction. The yield of 3-(2-naphthyl)-*D*-alanylhydantoic acid was 110 mg (0.43 mmol, 93%), which was all used in the second reaction with a volume of 95-ml 2 M HCl to give 82 mg (0.34 mmol) of **[2-^{14}C]-L-NMH** with a chemical yield of 79%. 2.0 mg of the crystals gave ^{14}C counts (Packard Tri-Carb 2100TR) with an average of 359954 dpm, which is equivalent to 6.00 kBq or 0.162 μCi; therefore, 82 mg of the crystals has a total of 6.65 μCi and a ^{14}C specific activity of 19.48 μCi/mmol. The [^{14}C]potassium cyanate used in the reaction had 500 μCi; hence the radiochemical yield was 6.65 μCi/500 μCi x 100 = 1.3%.

(Z)-5-(3-Bromobenzylidene)imidazolidine-2,4-dione (Thenmozhiyal et al, 2004) **(BVH):** This compound was synthesized according to the procedure of General method A (23%).

5-(1H-Indol-3-ylmethyl)imidazolidine-2,4-dione (Fei C, 2004) **(D/L-IMH):** This compound was synthesized from hydantoin and the relevant aldehyde according to the procedures of general methods A and B (76%). **mp** 220-223 °C. **TLC** R_f 0.05 (Petrol/EtOAc; 1:1). **^{1}H NMR** (300 MHz, DMSO-d_6) δ = 10.92 (1H, br s), 10.40 (1H, br s), 7.86 (1H, s), 7.55 (1H, d, *J* = 8.1), 7.30 (1H, d, *J* = 8.1), 7.10 (1H, s), 7.04 (1H, t, *J* = 7.3), 6.94 (1H, t, *J* = 7.3), 4.32 (1H, t, *J* = 4.5), 3.10 (2H, d, *J* = 4.5). **^{13}C NMR** (75 MHz, DMSO-d_6) δ = 175.7, 157.4, 135.9, 127.5, 124.1, 120.8, 118.6, 118.3, 111.2, 108.0, 58.3, 26.5. **IR** ν_{max}/cm^{-1} (neat) 3394, 3322, 3226, 2920, 2813, 2703, 2517, 1997, 1914, 1725, 1616, 1555. **ESI-MS** *m/z* 252.1 (100% MNa^+); (Found MNa^+, 252.0716 $C_{12}H_{11}N_3O_2$ requires *MNa* 252.0743).

(S)-5-((1H-Indol-3-yl)methyl)imidazolidine-2,4-dione (L-IMH): This compound was synthezised according to the procedure of Patching (Patching, 2011).

[2-^{14}C]-(S)-5-((1H-Indol-3-yl)methyl)imidazolidine-2,4-dione ([2-^{14}C]-L-IMH): This compound was synthesized according to the procedure of Patching (Patching, 2011).

(R)-5-((1H-indol-3-yl)Methyl)imidazolidine-2,4-dione (D-IMH): This compound was synthezised according to the procedure of Patching (Patching, 2011) to give 123 mg, 0.54 mmol, 54% from D-tryptophan) as colourless fine needles. **mp** ~ 220 °C. **^{1}H NMR** (500 MHz, DMSO-d_6) δ = 10.86 (s, 1H, N3-H), 10.32 (s, 1H, N1-H), 7.85 (s, 1H, indole-NH), 7.53 (d, 1H, *J* = 8.0 Hz, indole-H), 7.31 (d,

1H, *J* = 8.0 Hz, indole-H), 7.11 (d, 1H, *J* = 3.0 Hz, indole-H2), 7.05-6.94 (m, 2H, 2-indole-H), 4.29 (t, 1H, *J* = 5.0 Hz, H-5), 3.05 (d, 2H, *J* = 5.0 Hz, CH_2). **^{13}C NMR** (125 MHz, DMSO-d_6) δ = 175.7 (C-2), 157.4 (C-4), 135.9 (indole-C), 127.5 (indole-C), 124.1 (indole-C2), 120.8 (indole-C), 118.6 (indole-C), 118.3 (indole-C), 111.2 (indole-C), 108.0 (indole-C1), 58.3 (C-5), 26.5 (CH_2). **ESI-MS** (ESI^+/TOF) m/z calcd for $C_{12}H_{11}N_3O_2$ + H^+ = 230.0924; found: 230.0925 and calcd for $C_{12}H_{11}N_3O_2$ + Na^+ = 252.0743; found: 252.0734. **Elemental** Anal. calcd for $C_{12}H_{11}N_3O_2$: C, 62.88; H, 4.84; N, 18.33. Found: C, 62.80; H, 4.80; N, 18.35.

(S)-5-Benzylimidazolidine-2,4-dione (L-BH): This compound was synthesized according to the procedure of Patching (Patching, 2011).

(R)-5-Benzylimidazolidine-2,4-dione (D-BH): This compound was synthesized according to the procedure of Patching (Patching, 2011) to give 357 mg (2.33 mmol, 38% from D-phenylalanine) as colourless plates. **mp** 188-190 °C. **^{1}H NMR** (300 MHz, DMSO-d_6) δ = 10.40 (s, 1H, H-3), 7.89 (s, 1H, H-1), 7.32–7.20 (m, 5 H, Ar-H), 4.31 (t, 1H, $J_{H5,H6}$ = 5.0 Hz, H-5), 2.92 (m, 2H, *J* = 5.0 Hz, 14.0 Hz, 21.0 Hz, CH_2). **^{13}C NMR** (125 MHz, DMSO-d_6) δ = 175.1 (C-2), 157.1 (C-4), 135.6 (C-7), 129.7 (C-9), 128.1 (C-8), 126.6 (C-10), 58.4 (C-5), 36.4 (C-6). **ESI-MS** *m/z* calcd for $C_{10}H_{10}N_2O_2$ + H^+ = 191.0815; found: 191.0819 and calcd for $C_{10}H_{10}N_2O_2$ + Na^+ = 213.0634; found: 213.0631. **Elemental** Anal. calcd for $C_{10}H_{10}N_2O_2$: C, 63.15; H, 5.30; N, 14.73. Found: C, 63.15; H, 5.25; N, 14.90.

[^{14}C]-5-Ureidoimidazolidine-2,4-dione ([^{14}C-D/L-allantoin]): A ^{14}C-labelled version of this compound was synthesized according to the procedure of Patching (Patching, 2009).

References

Adams PD, Afonine PV, Bunkoczi G, Chen VB, Davis IW, Echols N, Headd JJ, Hung L-W, Kapral GJ, Grosse-Kunstleve RW, McCoy AJ, Moriarty NW, Oeffner R, Read RJ, Richardson DC, Richardson JS, Terwilliger TC, Zwart PH (2010) PHENIX: a comprehensive Python-based system for macromolecular structure solution. *Acta Crystallogr D* **66:** 213-221

Berendsen HJC, Postma JPM, DiNola A, Haak JR (1984) Molecular dynamics with coupling to an external bath. *J Chem Phys* **81:** 3684-3690

Bussi G, Donadio D, Parrinello M (2007) Canonical sampling through velocity rescaling. *J Chem Phys* **126:** 01410

Collaborative Computational Project Number 4 (1994) The CCP4 suite: programs for protein crystallography. *Acta Crystallogr D* **50:** 760-763

Delano WL (2002) The PyMOL Molecular Graphics System. *DeLano Scientific, Palo Alto, CA, USA*

Domański J, Stansfeld P, Sansom M, Beckstein O (2010) Lipidbook: A Public Repository for Force-Field Parameters Used in Membrane Simulations. *Journal of Membrane Biology* **236:** 255-258

Drew D, Lerch M, Kunji E, Slotboom DJ, de Gier JW (2006) Optimization of membrane protein overexpression and purification using GFP fusions. *Nat Methods* **3:** 303-313

Emsley P, Cowtan K (2004) Coot: model-building tools for molecular graphics. *Acta Crystallogr D* **60:** 2126-2132

Essman U, Perela L, Berkowitz ML, Darden T, Lee H, Pedersen LG (1995) A smooth particle mesh Ewald method. *J Chem Phys* **103:** 8577-8592

Fei C PW, Jianliang Z, Wei Q, Pingkai O (2004) The preparation of 3-(indolmethyl)-hydantoin. I. The hydrogenation process of 3-(indolmethylene)-hydantoin and separation of raney-Ni with magnetic material. . *Chemical reaction engineering and technology* **20:** 223-228

Henderson PJF, Macpherson AJS (1986) Assay, genetics, proteins, and reconstitution of proton-linked galactose, arabinose, and xylose transport-systems of escherichia-coli. *Methods in Enzymology* **125:** 387-429

Hess B (2008) P-LINCS: A Parallel Linear Constraint Solver for Molecular Simulation. *J Chem Theo Comp* **4:** 116-122

Hess B, Kutzner C, van der Spoel D, Lindahl E (2008) GROMACS 4: Algorithms for Highly Efficient, Load-Balanced, and Scalable Molecular Simulation. *J Chem Theo Comp* **4:** 435-447

Iorga B, Beckstein O (unpublished data)

Jensen KP, Jorgensen WL (2006) Halide, Ammonium, and Alkali Metal Ion Parameters for Modeling Aqueous Solutions. *J Chem Theo Comp* **2:** 1499-1509

Jones TA, Kjeldgaard M, Charles WC, Jr., Robert MS (1997) Electron-density map interpretation. *Methods in Enzymology* **277:** 173-208

Jorgensen WL, Madura JD (1985) Temperature and size dependence for Monte-Carlo simulations of TIP4P water. *Mol Phys* **56:** 1381-1392

Kabsch W (2010) XDS. *Acta Crystallogr D* **66:** 125-132

Kaminski GA, Friesner RA, Tirado-Rives J, Jorgensen WL (2001) Evaluation and Reparametrization of the OPLS-AA Force Field for Proteins via Comparison with Accurate Quantum Chemical Calculations on Peptides. *J Phys Chem B* **105:** 6474-6487

Kawaguchi T, Ueda M, Sashita R. (2000) D-3-(2-naphthyl)alanine manufacture with microorganism. Patent.

Kleinpeter E (1997) The structure of hydantoins in solution and in the solid state. *Structural Chemistry* **8:** 161-173

Kleywegt GJ, Jones TA (1994) A super position. *ESF/CCP4 Newsletter* **31:** 9

McCoy AJ, Grosse-Kunstleve RW, Adams PD, Winn MD, Storoni LC, Read RJ (2007) Phaser crystallographic software. *J Appl Crystallogr* **40:** 658-674

Michaud-Agrawal N, Denning EJ, Woolf TB, Beckstein O (2011) MDAnalysis: A Toolkit for the Analysis of Molecular Dynamics Simulations. *J Comp Chem* **32:** 2319–2327

Miyamoto S, Kollman PA (1992) SETTLE: An Analytical Version of the SHAKE and RATTLE Algorithms for Rigid Water Models. *J Comp Chem* **13:** 952-962

Otwinowski Z, Minor W, Charles W. Carter, Jr. (1997) Processing of X-ray diffraction data collected in oscillation mode. *Methods in Enzymology* **276:** 307-326

Patching SG (2009) Synthesis of highly pure 14C-labelled DL-allantoin and 13C NMR analysis of labelling integrity. *Journal of Labelled Compounds and Radiopharmaceuticals* **52:** 401-404

Patching SG (2011) Efficient syntheses of 13C- and 14C-labelled 5-benzyl and 5-indolylmethyl L-hydantoins. *Journal of Labelled Compounds and Radiopharmaceuticals* **54:** 110-114

Potterton L, McNicholas S, Krissinel E, Gruber J, Cowtan K, Emsley P, Murshudov GN, Cohen S, Perrakis A, Noble M (2004) Developments in the CCP4 molecular-graphics project. *Acta Crystallogr D* **60:** 2288-2294

Rizzo RC, Jorgensen WL (1999) OPLS all-atom model for amines: Resolution of the amine hydration problem. *J Am Chem Soc* **121:** 4827-4836

Sanchez-Weatherby J, Bowler MW, Huet J, Gobbo A, Felisaz F, Lavault B, Moya R, Kadlec J, Ravelli RB, Cipriani F (2009) Improving diffraction by humidity control: a novel device compatible with X-ray beamlines. *Acta crystallographica Section D, Biological crystallography* **65:** 1237-1246

Schaffner W, Weissmann C (1973) A rapid, sensitive, and specific method for the determination of protein in dilute solution. *Anal Biochem* **56:** 502-514

Shimamura T, Weyand S, Beckstein O, Rutherford NG, Hadden JM, Sharples D, Sansom MSP, Iwata S, Henderson PJF, Cameron AD (2010) Molecular Basis of Alternating Access Membrane Transport by the Sodium-Hydantoin Transporter Mhp1. *Science* **328:** 470-473

Shimamura T, Yajima S, Suzuki S, Rutherford NG, O'Reilly J, Henderson PJ, Iwata S (2008) Crystallization of the hydantoin transporter Mhp1 from Microbacterium liquefaciens. *Acta Crystallogr Sect F Struct Biol Cryst Commun* **64:** 1172-1174

Studier FW (2005) Protein production by auto-induction in high density shaking cultures. *Protein Expr Purif* **41:** 207-234

Suzuki S, Henderson PJF (2006) The hydantoin transport protein from Microbacterium liquefaciens. *Journal of Bacteriology* **188:** 3329-3336

Takuma Y, Watanabe N. (2001) Preparation of 5-(substituted naphthylmethyl)hydantoins. Patent.

Thenmozhiyal JC, Wong PTH, Chui WK (2004) Anticonvulsant activity of phenylmethylenehydantoins: A structure-activity relationship study. *Journal of Medicinal Chemistry* **47:** 1527-1535

Ulmschneider JP, Ulmschneider MB (2009) United atom lipid parameters for combination with the OPLS-AA force field. *J Chem Theo Comp* **5:** 1803–1813

Wagner S, Klepsch MM, Schlegel S, Appel A, Draheim R, Tarry M, Hogbom M, van Wijk KJ, Slotboom DJ, Persson JO, de Gier JW (2008) Tuning Escherichia coli for membrane protein overexpression. *Proc Natl Acad Sci U S A* **105:** 14371-14376

Ward A, Sanderson NM, O'Reilly J, Rutherford NG, Poolman B, Henderson PJF (2000) *The amplified expression, identification, purification, assay and properties of hexahistidine-tagged bacterial membrane transport proteins, in Membrane Transport – A Practical Approach* Oxford: Blackwell's Press.

Weyand S, Shimamura T, Yajima S, Suzuki S, Mirza O, Krusong K, Carpenter EP, Rutherford NG, Hadden JM, O'Reilly J, Ma P, Saidijam M, Patching SG, Hope RJ, Norbertczak HT, Roach PC, Iwata S, Henderson PJ, Cameron AD (2008) Structure and molecular mechanism of a nucleobase-cation-symport-1 family transporter. *Science* **322:** 709-713

Winn MD, Isupov MN, Murshudov GN (2001) Use of TLS parameters to model anisotropic displacements in macromolecular refinement. *Acta Crystallogr D* **57:** 122-133

Winter G (2010) xia2: an expert system for macromolecular crystallography data reduction. *Journal of Appl Crystallogr* **43:** 186-190

Witholt B, Boekhout M, Brock M, Kingma J, Heerikhuizen HV, Leij LD (1976) An efficient and reproducible procedure for the formation of spheroplasts from variously grown Escherichia coli. *Anal Biochem* **74:** 160-170
