# Supplementary material for: Molecular mechanism of ligand recognition by membrane transport protein, Mhp1
Source: EMBO J. 2014 Jun 21;33(16):1831–44. doi: 10.15252/embj.201387557 (PMC4195764; doi:10.15252/embj.201387557)
Supplement: Supplementary file 12 [file embj0033-1831-sd12.pdf]

THE EMBO JOURNAL

Manuscript EMBO-2013-87557

Molecular mechanism of ligand recognition by membrane transport protein, Mhp1

Katie J Simmons, Scott M Jackson, Florian Brueckner, Simon G Patching, Oliver Beckstein, Ekaterina Ivanova, Tian Geng, Simone Weyand David Drew, Joseph Lanigan, David J Sharples, Mark SP Sansom, So Iwata, Colin WG Fishwick, A Peter Johnson, Alexander D Cameron and Peter JF Henderson

Corresponding author: Peter Henderson, University of Leeds and Colin Fishwick, University of Warwick

Review timeline:

Submission date:	03 December 2013
Editorial Decision:	17 January 2014
Revision received:	01 April 2014
Editorial Decision:	27 April 2014
Revision received:	07 May 2014
Accepted:	14 May 2014

Transaction Report:

(Note: With the exception of the correction of typographical or spelling errors that could be a source of ambiguity, letters and reports are not edited. The original formatting of letters and referee reports may not be reflected in this compilation.)

Editor: Anne Nielsen

1st Editorial Decision 17 January 2014

Thank you for submitting your manuscript for consideration by the EMBO Journal and my apologies for the unusually long duration of the review process in this case. Your study has now been seen by two referees whose comments are shown below.

As you will see from the reports both referees highlight the importance of your findings and recommend publication of your manuscript in The EMBO Journal following appropriate - and extensive - revision. In addition to raising a number of specific technical points on data presentation, modeling and interpretation of density that will all have to be addressed in full in a revised manuscript, both referees encourage you to extensively rewrite the manuscript in order to more clearly convey the merit of the reported findings in light of the existing literature. We would especially ask you to clarify all issues arising from model refinement as pointed out by both refs (ref #1 point 6, 7 and ref #2 points 2, 8, 9) and to strengthen the biochemical analysis of mutant proteins as requested by ref #2 (points 3, 5, 6, 7).

Given the referees' overall positive recommendations, I would invite you to submit a revised version of the manuscript, addressing all criticisms raised by both referees. I should add that it is EMBO Journal policy to allow only a single round of revision, and acceptance or rejection of your manuscript will therefore depend on the completeness of your responses in this revised version.

When preparing your letter of response to the referees' comments, please bear in mind that this will form part of the Review Process File, and will therefore be available online to the community. For more details on our Transparent Editorial Process, please visit our website: http://www.nature.com/emboj/about/process.html

We generally allow three months as standard revision time. As a matter of policy, competing manuscripts published during this period will not negatively impact on our assessment of the conceptual advance presented by your study. However, we request that you contact the editor as soon as possible upon publication of any related work, to discuss how to proceed. Should you foresee a problem in meeting this three-month deadline, please let us know in advance and we may be able to grant an extension.

Thank you for the opportunity to consider your work for publication. I look forward to your revision.

REFEREE REPORTS:

Referee #1:

The manuscript presents the structure of Mhp1 in complex with L-IMH at 3.4 ≈, giving insights into more details in the ligand interactions with MhpI. Subsequent structural analysis of BVH-MhpI and BH-MhpI complexes indicated that each ligand adopts the extended form in the binding pocket with the hydantoin ring on the deepest in the pocket, whereas BH was previously modeled as the U-shaped configuration in the complex structure. Molecular dynamics simulations also supported the extended forms of the substrates in the binding pocket. Mutation analysis revealed the importance of hydrogen bonding between MhpI and the hydantoin moiety of the substrate. In addition, 14C-L-IMH transport inhibition by a series of 5-substituted hydantoin molecules revealed that hydrophobic moiety at this position is also required for effective binding to MhpI. Further analysis has led to the finding of a novel inhibitor NMH, which has a bulky substituent and a higher affinity to MhpI. The crystal structure of NMH-MhpI complex represents a similar structure with the IMH-bound state, but with TMH10 (the extracellular thin gate) remaining in the open position. This structure suggested that the steric hindrance between the bulky substituent and Leu363 on TMH10 interferes with the transition toward the occluded state by the inward movement of TMH10. This is also supported by the Leu363Ala mutation restoring the NMH transport activity. Based on the crystal structures of MhpI in complex with L-IMH, L-BH, and NMH, molecular dynamics simulations, mutagenesis, and binding and transporting activity for the synthesized ligands, authors proposed a molecular mechanism of MhpI for ligand recognition and sequential conformational transition toward the occluded state.

The proposed mechanism in transition toward the occluded state is different from that have been proposed for LeuT, although they share similarities in the overall architecture and transport cycle in the 5HIRT family. Such differences in the ligand recognition and the following conformational changes are of importance, however too much results stuffed in the manuscript made it uneasy to understand the substances.

Major points

1. There are so many "see below" and "described below" in the manuscript, making it difficult to follow the contexts. In addition, there is no discussion below in some cases (related with the Major point 5). Figures are not kind for interpreting the manuscript. For example in Figure 1a, there is no numbering for Gly219, and an error numbering for W220 (W22 in the figure).

2. Mutation of G219 to Ser or Ile does not support the hydrogen bonding between the G219 carbonyl oxygen and L-IMH, because, as described in the manuscript, these mutations would result

in a clash of the side chain with the indole ring of L-IMH. ("Consistently, its replacement with serine or isoleucine reduced both binding and transport activity" at the bottom of p.9)

3. The manuscript mostly describes about ligand recognition in MhpI, which are not general in other members of 5HIRT. Therefore the title should clearly shows that it reports on "ligand recognition in MhpI".

4. No error bars are attached on Supplementary Figure S4, although the explanation about error bars is present in the figure legend.

5. Despite the sentence "approximately 70% in the conformation of the occluded structure and 30% in the conformation of the open structure. The significance of this will be discussed later" in p.8, any other discussion are present in the main text.

6. The refined structure for BVH-MhpI complex contains much noises in both 2mFo-DFc and mFo-Fc maps for TMH2 as well as TMH10, thus not seemingly supporting the conformational heterogeneity of TMH10 discussed in the manuscript (Supplementary Figure S7b).

7. The electron density map for TMH10 in NMH-MhpI complex structure should be shown, because the different orientation of TMH10 in this structure is the most of importance in this manuscript.

8. The inhibitory effect of the racemic NMH is not between those of the enantiomers (D-NMH and L-NMH). It is possible that contaminants or the purity of the synthesized chemicals affect the transport activity. In this case, it is not adequate for describing "For NMH, however, the two enantiomers bind with similar affinities" in p.12.

Minor points

1. "It appears to be oriented by two hydrogen bonds from the side chain of Asn318, and another to the carbonyl oxygen of Gly219 at the break of TMH6 in the bundle motif" in p.6, this sentence is not kind to readers.

2. The word "open-out" in p.7, which describes a state of transporters, is not general and inconsistent with "outward-open" in the other parts of the manuscript.

3. In the figure legends for S1 and S7, symbol font for "sigma" is not appropriately embedded in the pdf files, resulted in unreadable characters. (also same in "micro" in S4)

4. Numbers in the middle of Supplementary Table SI in italic should be written in block style. The index "c" beside Rfree in the same table should be corrected to "b".

Referee #2:

Simmons, Jackson, Brueckner, Patching et al. report a comprehensive analysis of ligand-bound complexes of Mhp1 - an established model of the important nucleobase-cation symporter family, and one of the pioneering models of the insight gained into the alternating access transport mechanisms related to the LeuT-fold/5HIRT superfamily of active, secondary transporters. Combining low-resolution crystallography (3.4 - 3.9 ≈ max. resolution) with MD simulations, Trp fluorimetric analysis of ligand binding and whole-cell accumulation measurements of transport the activity of wt and mutant forms and series of substrate derivatives are analyzed. An extended conformation of substrates is observed (earlier modeled as U-shaped), TM10 is implicated in substrate occlusion, and particular residues are pinpointed for substrate recognition, in particular Asn318, Gln121 and Gly219 for hydantoin binding and Trp117/Trp220 for sandwiching of the extended hydrophobic substituent.
The experiments are generally well performed, the data are conclusive and yield important new insight and refined models. The following are therefore benign remarks on how possibly to improve an important contribution.

Major points:

1. Only at page 15 appears a small note on Na+. To the general reader it should be made more clear in the introduction how Mhp1 is a Na+ dependent transporter and how substrate binding occurs in Na+dependent manner. Also in the discussion the coupled function of Na+ binding, substrate binding and transport should be addressed in the interpretation of the mutational data

2. The high R-factors for the lower resolution complexes are not comforting. The 3.4 ≈ structure seems very reasonable at state of the art, but eg. an Rfree at 33.7% for the L-BH complex is surprisingly high in comparison with a reliable and related higher res structure being available. The maps are certainly telling (Fig. S1), but the low-res refinements may have introduced errors in the interpretations. Any twinning issues? In that context it would be nice also to see from table S1 the space group and contents of the asymmetric unit - I can find it in cited papers, but should appear directly from table S1. Furthermore, the resolution of the crystallographic data should be qualified by CC-0.5 and R/Rfree for the outer resolution bins (table S1). These current issues indicate that the structures do not represent trivial reproduction, and Table S1 therefore also ought to go into the main paper so that the reader more immediately grasps a proper level of confidence.

3. One would generally request duplicate/triplicates of protein expression/purification for binding studies to confirm that observed effects/trends on mutants are not merely due to batch-to-batch variations (this level of reproduction could be limited to wt and some of the mutant forms, e.g. validating "trend series")

Minor points:

4. Since the structures and MD analyses stem from low resolution crystallographic data It would be helpful to associate the introductory remark on page 7 ("We then examined the conformational changes...") with a general disclaimer, such as "It should be noted that the reported distances will be associated with significant inaccuracies due to the low resolution of the reported structures".

5. The Gly219 mutants (Ser, Ile) are not surprisingly showing an effect. The Ser mutant likely perturbs the backbone and a Pro also should show a dramatic effect here - exceeding that of Ile. Furthermore, the smallest possible substitution (Ala) would seem important to include to understand the Ser vs. Ile data (particular Gly flexibility or mere size important?).

6. The Gln42 data are not conveyed well - page 10 should be rewritten and seems biased by the structures/MD, whereas the functional data indicate that it is quite important. Could it be important in other intermediate states than those discussed here?

7. A possible role of Trp220 in Na+ coupling? - do we fail to see an effect of Trp220 mutants on ligand binding/transport because assay conditions don't provoke it? E.g. Na+ dependence of transport kinetics? - And how do Trp220 mutants respond to the napthalyl substituent and/or other substrate variations?

8. How was the NMH complex refined - as a 0.5:0.5 mixture of D- and L-complexes? Whichever way, is it a reasonable model based on binding affinities? Shoudl be described

9. Similarly, how were the alternating conformations of TM10 for different complexes validated in refinement - significant improvements in Rfree? The validation criteria should be described

10. Table I - the header "Uptake (%)" should be changed to reflect properly the competetion assay in question - at first glance one would think that 100% means excellent uptake properties.

11. What happens at the end of the simulation in Fig. S3j?

1st Revision - authors' response 01 April 2014

Response to both referees and editors comments.

Editors and Referees comments are in roman type face. Our comments are in italics.

As you will see from the reports both referees highlight the importance of your findings and recommend publication of your manuscript in The EMBO Journal following appropriate - and extensive - revision. In addition to raising a number of specific technical points on data presentation, modelling and interpretation of density that will all have to be addressed in full in a revised manuscript, both referees encourage you to extensively rewrite the manuscript in order to more clearly convey the merit of the reported findings in light of the existing literature. We would especially ask you to clarify all issues arising from model refinement as pointed out by both refs (ref #1 point 6, 7 and ref #2 points 2, 8, 9) and to strengthen the biochemical analysis of mutant proteins as requested by ref #2 (points 3, 5, 6, 7).

Given the referees' overall positive recommendations, I would invite you to submit a revised version of the manuscript, addressing all criticisms raised by both referees. I should add that it is EMBO Journal policy to allow only a single round of revision, and acceptance or rejection of your manuscript will therefore depend on the completeness of your responses in this revised version.

We thank the Referees and the Editor for their overall approval of the substance of our paper and for their insightful suggestions for improvements. We have acted on all these as detailed below. In addition we have made many modifications throughout the text in order to convey more clearly the merit of the reported findings.-We have simplified the paper (as suggested by Referee 1) by omitting compounds that were only minor chemical modifications to the main ligands discussed and/or had little effect on transport of L-IMH or binding of L-BH. We have also amplified the discussion of the comparison of Mhp1 with other LeuT family members as suggested by the editor.

Referee #1:

The manuscript presents the structure of Mhp1 in complex with L-IMH at 3.4 Å, giving insights into more details in the ligand interactions with MhpI. Subsequent structural analysis of BVH-MhpI and BH-MhpI complexes indicated that each ligand adopts the extended form in the binding pocket with the hydantoin ring on the deepest in the pocket, whereas BH was previously modeled as the U-shaped configuration in the complex structure. Molecular dynamics simulations also supported the extended forms of the substrates in the binding pocket. Mutation analysis revealed the importance of hydrogen bonding between MhpI and the hydantoin moiety of the substrate. In addition, 14C-L-IMH transport inhibition by a series of 5-substituted hydantoin molecules revealed that hydrophobic moiety at this position is also required for effective binding to MhpI. Further analysis has led to the finding of a novel inhibitor NMH, which has a bulky substituent and a higher affinity to MhpI. The crystal structure of NMH-MhpI complex represents a similar structure with the IMH-bound state, but with TMH10 (the extracellular thin gate) remaining in the open position. This structure suggested that the steric hindrance between the bulky substituent and Leu363 on TMH10 interferes with the transition toward the occluded state by the inward movement of TMH10. This is also supported by the Leu363Ala mutation restoring the NMH transport activity. Based on the crystal structures of MhpI in complex with L-IMH, L-BH, and NMH, molecular dynamics simulations, mutagenesis, and binding and transporting activity for the synthesized ligands, authors proposed a molecular mechanism of MhpI for ligand recognition and sequential conformational transition toward the occluded state. The proposed mechanism in transition toward the occluded state is different from that have been proposed for LeuT, although they share similarities in the overall

architecture and transport cycle in the 5HIRT family. Such differences in the ligand recognition and the following conformational changes are of importance, however too much results stuffed in the manuscript made it uneasy to understand the substances.

We thank the referee for the insightful analysis and positive comments. In order to address this last point of "too many results stuffed in the manuscript" we have removed from the text descriptions of the effects of minor chemical modifications to the hydantoin on ligand binding. The text now focuses on those compounds that were effective ligands and a few judiciously chosen ineffective controls. These are all in Table 2, and the large Table of compounds is consequently removed from the Supplementary Information. We believe that the text now flows much more easily and is less confusing.

Major points

1. There are so many "see below" and "described below" in the manuscript, making it difficult to follow the contexts. In addition, there is no discussion below in some cases (related with the Major point 5). Figures are not kind for interpreting the manuscript. For example in Figure 1a,there is no numbering for Gly219, and an error numbering for W220 (W22 in the figure).

We have made many changes to the text to improve the continuity and the cross-referencing. Figure 1a does not number any individual residues, and we think the referee means Figure 1b. The omission of the zero from W220 (and of the nine from Asn319) occurred during transmission of the digital data. Figures will be uploaded differently in the revised manuscript.

2. Mutation of G219 to Ser or Ile does not support the hydrogen bonding between the G219 carbonyl oxygen and L-IMH, because, as described in the manuscript, these mutations would result in a clash of the side chain with the indole ring of L-IMH. ("Consistently, its replacement with serine or isoleucine reduced both binding and transport activity" at the bottom of p.9)

We apologise for this error, which was pointed out by both referees. By omitting "consistently", which we inadvertently added during the editing process, the paragraph reads as it was originally intended. We have now modified the paragraph to make it clearer.

3. The manuscript mostly describes about ligand recognition in MhpI, which are not general in other members of 5HIRT. Therefore the title should clearly shows that it reports on "ligand recognition in MhpI".

The title has been altered to:

"The molecular mechanism of ligand recognition by the membrane transport protein, Mhp1"

4. No error bars are attached on Supplementary Figure S4, although the explanation about error bars is present in the figure legend.

This has been corrected.

5. Despite the sentence "approximately 70% in the conformation of the occluded structure and 30% in the conformation of the open structure. The significance of this will be discussed later" in p.8, any other discussion are present in the main text.

"The significance of this will be discussed later" has been removed.

6. The refined structure for BVH-MhpI complex contains much noises in both 2mFo-DFc and mFo-Fc maps for TMH2 as well as TMH10, thus not seemingly supporting the conformational heterogeneity of TMH10 discussed in the manuscript (Supplementary Figure S7b).

We agree with this comment. To show this in the BVH-Mhp1 density maps the contour level needs to be set very low. It is much more apparent in another compound with iodine replacing the bromine but we do not show these data. On consideration we have decided to omit the second minor conformation from the final structure and instead put a remark in the header of the PDB file. This is described in the Supplementary Methods. The short discussion on this was an "interesting observation" but may detract rather than add anything to the paper. Our real intention with the inclusion of the BVH-Mhp1 crystal structure was to use the anomalous signal of the bromine to confirm the orientation of the compound.

7. The electron density map for TMH10 in NMH-MhpI complex structure should be shown, because the different orientation of TMH10 in this structure is the most of importance in this manuscript.

The map was already shown in Supplementary Figure S7b as discussed for point 6. We have decided to redo this figure and put in the electron density for the final map as well. The figure shows clearly that TMH10 is in the open position. At this resolution the exact position of the side chains are more ambiguous but our mutation of Leu363 supports our position.

8. The inhibitory effect of the racemic NMH is not between those of the enantiomers (D-NMH and L-NMH). It is possible that contaminants or the purity of the synthesized chemicals affect the transport activity. In this case, it is not adequate for describing "For NMH, however, the two enantiomers bind with similar affinities" in p.12.

We understand where the referee is coming from, but the day-to-day variability of the uptake dose-response curves is sufficient that the failure of the curve for the racemic mixture not to fall between those of each enantiomer may or may not be significant. We have added the phrase "that are indistinguishable within the experimental error" in the text to clarify this. The purity of the synthesised compounds was generally assessed by both MS and NMR, and if contaminants were detected they were reduced by recrystallisations(s).

Minor points

1. "It appears to be oriented by two hydrogen bonds from the side chain of Asn318, and another to the carbonyl oxygen of Gly219 at the break of TMH6 in the bundle motif" in p.6, this sentence is not kind to readers.

This paragraph has been modified.

© EMBO

2. The word "open-out" in p.7, which describes a state of transporters, is not general and inconsistent with "outward-open" in the other parts of the manuscript.

All uses have been corrected to outward-open.

3. In the figure legends for S1 and S7, symbol font for "sigma" is not appropriately embedded in the pdf files, resulted in unreadable characters. (also same in "micro" in S4)

These errors arise during transmission of the digital text, and may have to be corrected by the typesetters should the error persist on resubmission.

4. Numbers in the middle of Supplementary Table SI in italic should be written in block style. The index "c" beside Rfree in the same table should be corrected to "b".

This table has been corrected accordingly and also modified as requested by Referee 2.

Referee #2:

Simmons, Jackson, Brueckner, Patching et al. report a comprehensive analysis of ligand-bound complexes of Mhp1 - an established model of the important nucleobase-cation symporter family, and one of the pioneering models of the insight gained into the alternating access transport mechanisms related to the LeuT-fold/5HIRT superfamily of active, secondary transporters. Combining low-resolution crystallography (3.4 - 3.9 Å max. resolution) with MD simulations, Trp fluorimetric analysis of ligand binding and whole-cell accumulation measurements of transport the activity of wt and mutant forms and series of substrate derivatives are analysed. An extended conformation of substrates is observed (earlier modeled as U-shaped), TM10 is implicated in substrate occlusion, and particular residues are pinpointed for substrate recognition, in particular Asn318, Gln121 and Gly219 for hydantoin binding and Trp117/Trp220 for sandwiching of the extended hydrophobic substituent. The experiments are generally well performed, the data are conclusive and yield important new insight and refined models. The following are therefore benign remarks on how possibly to improve an important contribution.

Major points:

1. Only at page 15 appears a small note on Na+. To the general reader it should be made more clear in the introduction how Mhp1 is a Na+ dependent transporter and how substrate binding occurs in Na+dependent manner.

We have added this in the first sentence and discuss it again at the end of the third paragraph.

Also in the discussion the coupled function of Na+ binding, substrate binding and transport should be addressed in the interpretation of mutational data.

We appreciate that it is reasonable to raise the issue of the role of Na^+ and have amplified our discussion in this regard. The role of Na^+ ions in Mhp1 appears to be to change the proportion of Mhp1 from an inward-facing conformation to the outward-open conformation necessary for the binding of hydantoin, consistent with other LeuT family members. The Na^+-site, does not change appreciably (at the limit of the resolution) as the hydantoin binds. Hence, though the acquisition of substrate/inhibitor is Na^+-dependent, the molecular events effecting acquisition and of specificity are independent of the role of the cation.

We have, in fact, a wealth of additional data on the effects of the Na+ ions, which is being incorporated into another substantial manuscript on the (non)steady state kinetics of the Na+ and hydantoin binding to the wild-type and proteins mutated in the Na^+-binding site. In the light of all these studies we believe that the molecular events and specificity of hydantoin binding to the outward-open form and its subsequent closure constitute a coherent story in themselves without introducing more material on the cation binding. The referees do seem largely to agree with this.

2. The high R-factors for the lower resolution complexes are not comforting. The 3.4 Å structure seems very reasonable at state of the art, but e.g. an Rfree at 33.7% for the L-BH complex is surprisingly high in comparison with a reliable and related higher res structure being available. The maps are certainly telling (Fig. S1), but the low-res refinements may have introduced errors in the interpretations. Any twinning issues? In that context it would be nice also to see from table S1 the space group and contents of the asymmetric unit - I can find it in cited papers, but should appear directly from table S1. Furthermore, the resolution of the crystallographic data should be qualified by CC-0.5 and R/Rfree for the outer resolution bins (table S1). These current issues indicate that the structures do not represent trivial reproduction, and Table S1 therefore also ought to go into the main paper so that the reader more immediately grasps a proper level of confidence.

Table S1 has now been modified and put in the main paper as requested (Table 1). The major issue with the data is that it is very anisotropic. With such data it is not always trivial to decide on a resolution cut-off. We decided on this based on where the CC-0.5 starts dropping quickly. We now quote the value in the table and also the resolution along the three crystallographic axes at which the CC-0.5 drops below 0.5. The structure in which the anisotropy (BH) was the worst is the one with the highest Rfree. We believe this value to be due to the bulk and anisotropy scaling algorithms of the refinement program. In the current version of phenix (1.8.4) the R-values are much more respectable for the same structure, data and refinement protocols, though the final maps are little different. The final refinement runs have now all used this newer version of Phenix. Notably the R-factors for the best data set (IMH) are little changed. We have now refined the NMH structure at 3.7Å rather than 3.9Å. As the referee points out, though the resolution of the data is low, the conclusions we draw are clear from the electron density maps.

3. One would generally request duplicate/triplicates of protein expression/purification for binding studies to confirm that observed effects/trends on mutants are not merely due to batch-to-batch variations (this level of reproduction could be limited to wt and some of the mutant forms, e.g. validating "trend series")

For the wild-type Mhp1 binding studies have been performed on over 30 biological preparations, with never less than three technical replicates 'on-the-day' and more on different days accumulated to yield the final s.e.m. value. For most of the mutants there were 1-2 biological preparations with three technical replicates 'on-the-day' and more on 2-3 different days, from all of which the given s.e.m. values were calculated. In no case were 'typical' values used.

Minor points:

4. Since the structures and MD analyses stem from low resolution crystallographic data It would be

helpful to associate the introductory remark on page 7 ("We then examined the conformational changes...") with a general disclaimer, such as "It should be noted that the reported distances will be associated with significant inaccuracies due to the low resolution of the reported structures".

We had thought our general tone was pitched to convey this message. However we have now added "We next examined the conformational changes that occur upon binding of ligands to Mhp1 and that can be interpreted at the modest resolution of the ligand complexes. "

5. The Gly219 mutants (Ser, Ile) are not surprisingly showing an effect. The Ser mutant likely perturbs the backbone and a Pro also should show a dramatic effect here - exceeding that of Ile. Furthermore, the smallest possible substitution (Ala) would seem important to include to understand the Ser vs. Ile data (particular Gly flexibility or mere size important?).

As discussed above for point 2 of Referee 1 the paragraph regarding Gly219 was misleading due to an error introduced during our editing of the manuscript. While these would be interesting mutations to do, as Referee 1 points out the manuscript is already result-heavy.

6. The Gln42 data are not conveyed well - page 10 should be rewritten and seems biased by the structures/MD, whereas the functional data indicate that it is quite important. Could it be important in other intermediate states than those discussed here?

We have now revised the text as suggested. It is possible that the residue affects sodium ion binding but this is beyond the scope of the paper.

7. A possible role of Trp220 in Na+ coupling? - do we fail to see an effect of Trp220 mutants on ligand binding/transport because assay conditions don't provoke it? E.g. Na+ dependence of transport kinetics? - And how do Trp220 mutants respond to the napthalyl substituent and/or other substrate variations?

We accept these points but prefer to cover them in another paper that includes a very substantial exploration of the effects of Na^{+} especially as Referee 1 seems rather against inclusion of more data.

8. How was the NMH complex refined - as a 0.5:0.5 mixture of D- and L-complexes? Whichever way, is it a reasonable model based on binding affinities? Should be described.

We apologise for not including this information in the original manuscript and have now added it. Essentially there is no discrimination in the assays between the L and D forms and at the resolution of the data both fit reasonably well. We therefore refined them as 0.5:0.5.

9. Similarly, how were the alternating conformations of TM10 for different complexes validated in refinement - significant improvements in Rfree? The validation criteria should be described

See reply to Referee 1 comment 6. Essentially we had modeled the alternating conformations based on the electron density. We have now replaced the alternating conformations with a comment in the header of the PDB file.

10. Table I - the header "Uptake (%)" should be changed to reflect properly the competition assay in question - at first glance one would think that 100% means excellent uptake properties.

Agreed and done.

11. What happens at the end of the simulation in Fig. S3j?

We extended the simulation from ~100ns to 500ns. The plot corresponding to S3j is now included in an updated Figure S3 in the Supplementary Information. The indole of IMH briefly flips and then returns to its previous state (the flip just occurred at the end of the simulation). All in all, the simulation behaves similarly to the one shown before.

2nd Editorial Decision 27 April 2014

Thank you for submitting a revised version of your manuscript. It has now been seen by the original referees who both find that all original criticisms raised have been adequately addressed and thus recommend your study for publication in The EMBO Journal.
Given the referees' positive recommendations, I would like to invite you to submit a final revised version of the manuscript, addressing the following editorial points:

-> Could you please include a paragraph stating all author contributions?
-> Please correct formatting issues in table 1 as pointed out by ref #1
-> Could you consider rephrasing the title along the lines of 'Molecular mechanism of ligand recognition by membrane transport protein Mhp1'?
-> Please provide information on nature of error bars and underlying statistics for figure 4 in the figure legend (as already done for fig 3)
-> Would it be possible to provide a higher-resolution version of fig 4?
-> Please make sure that all structural data obtained has been adequately deposited in public databases and referenced in the manuscript

I would also ask you to move the synthesis information depicted in fig 7 and 8 to a supplementary figure (thus reducing the total number of figures in the manuscript to 6), since these two figures address materials and methods rather than the main manuscript. Please let me know if you would disagree to this change.

As of Jan 1st 2014 every paper published in The EMBO Journal will include a 'Synopsis' to further enhance its discoverability. Synopses are displayed on the html version of the paper and are freely accessible to all readers. The synopsis will include a short standfirst - written by the handling editor - as well as 2-5 one sentence bullet points that summarise the paper and are provided by the authors. These bullet points should be complementary to the abstract - i.e. not repeat the same text. We encourage inclusion of key acronyms and quantitative information. I would therefore ask you to include your suggestions for bullet points.

Thank you again for your contribution to The EMBO Journal. I look forward to receiving your final revision.

REFEREE REPORTS

Referee #1:

The revised manuscript is now easily readable for general readers, with important notion in the

mechanism of MhpI. Based on the crystal structures, MD simulations, mutagenesis, and biochemical assay using synthesized chemicals, authors carefully verified the detailed mechanism for MhpI in this study, which would also advance understanding of molecular basis for other 5-helix inverted repeat transporters. Totally the authors are answering to all the points, but some errors should be corrected. For example, numbers for "I/ (I)" and "Rmerge" in Table 1 are in italic fonts.

Referee #2:

The organization and flow of the manuscript has greatly, and the points raised by the reviewers have been addressed well or sufficiently
